# Supplementary material for: Survival of Single Positive Thymocytes Depends upon Developmental Control of RIPK1 Kinase Signaling by the IKK Complex Independent of NF-κB
Source: Immunity. 2019 Feb 19;50(2):348–361.e4. doi: 10.1016/j.immuni.2019.01.004 (PMC6382466; doi:10.1016/j.immuni.2019.01.004)
Supplement: Document S1. Figure S1 [file mmc1.pdf]

Immunity, Volume *50*

Supplemental Information

Survival of Single Positive Thymocytes Depends upon Developmental Control of RIPK1 Kinase Signaling by the IKK Complex Independent of NF-κB

Louise V. Webb, Alessandro Barbarulo, Jelle Huysentruyt, Tom Vanden Berghe, Nozomi Takahashi, Steven Ley, Peter Vandenabeele, and Benedict Seddon

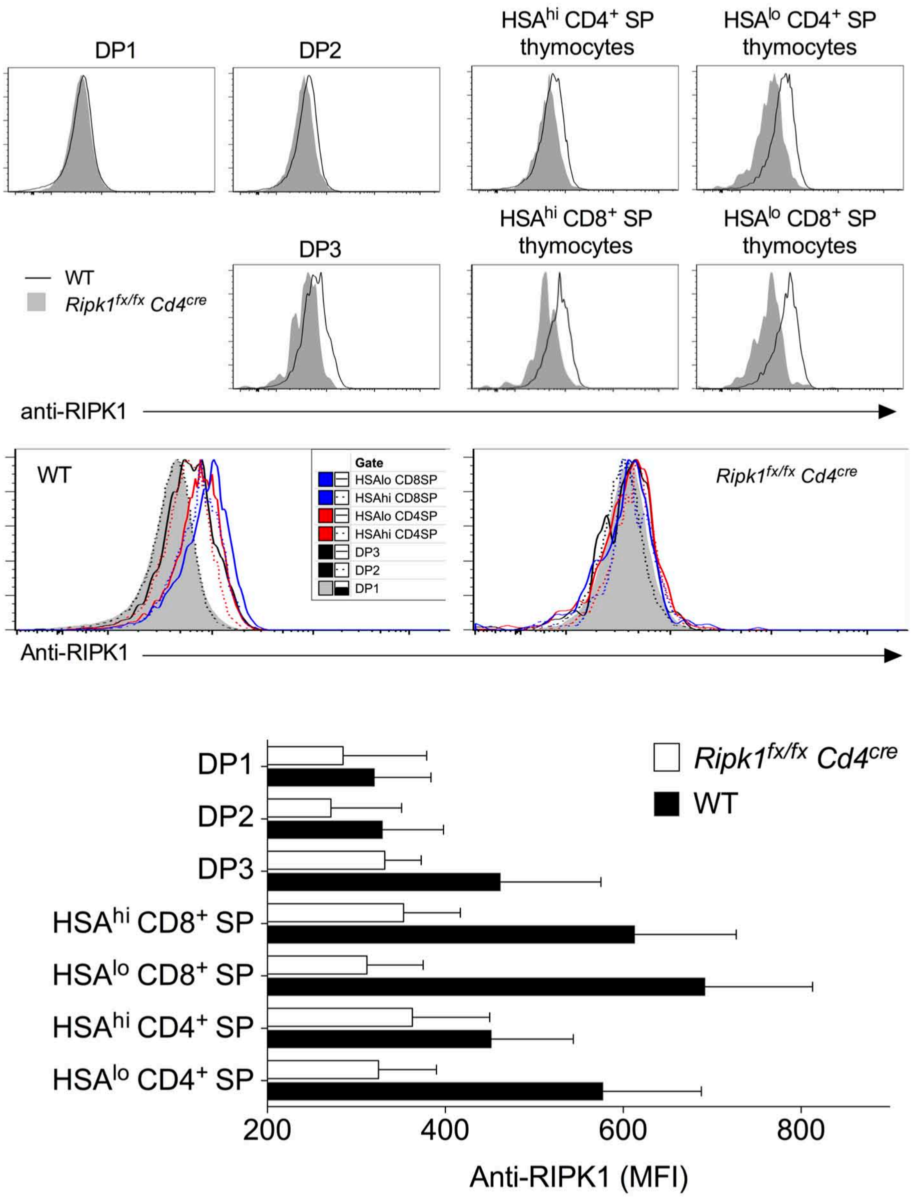

Figure S1 - RIPK1 expression in WT controls vs RIPK1 deficient thymocytes. Related to Figure 4. Thymocytes from 8 week old $CD4^{Cre}$ $Ripk1^{fx/fx}$ (n=3) and Cre– littermates (n=3) were isolated and stained for expression of CD4, CD8, TCR, CD5, HSA followed by fixation and intracellular stain for RIPK1 expression. Histograms show RIPK1 staining by thymocytes from Cre+ (RIPK1 KO) and Cre- (WT), comparing either RIPK1 staining of specific thymic subsets between Cre+ and Cre- mice (top two rows), or directly comparing staining of different subpopulations from Cre– (WT) and $CD4^{Cre}$ $Ripk1^{fx/fx}$ mice (bottom row). Subsets were gated as follows : DP1 ($CD4^{+}CD8^{+}$ $TCR^{LO}CD5^{LO}$), DP2 ($CD4^{+}CD8^{+}$ $TCR^{INT}CD5^{HI}$), DP3 ($CD4^{+}CD8^{+}$ TCR^{HI} $CD5^{INT}$), HSA^{hi} CD4SP ($CD4^{+}CD8^{-}$ $TCR^{HI}CD5^{HI}$ HSA^{HI}), HSA^{lo} CD4SP ($CD4^{+}CD8^{-}$ TCR^{HI} $CD5^{HI}$ HSA^{LO}), HSA^{hi} CD8SP ($CD4^{-}CD8^{+}$ $TCR^{HI}CD5^{HI}$ HSA^{HI}) and HSA^{lo} CD8SP ($CD4^{-}CD8^{+}$ $TCR^{HI}CD5^{HI}$ HSA^{LO}). Bar chart shows mean fluorescence intensity of RIPK1 staining on the indicated thymic subsets from either $CD4^{Cre}$ $Ripk1^{fx/fx}$ (RIPK1 KO) and Cre– (WT) littermates.
