# Supplementary material for: Survival of Single Positive Thymocytes Depends upon Developmental Control of RIPK1 Kinase Signaling by the IKK Complex Independent of NF-κB
Source: Immunity. 2019 Feb 19;50(2):348–361.e4. doi: 10.1016/j.immuni.2019.01.004 (PMC6382466; doi:10.1016/j.immuni.2019.01.004)
Supplement: Document S2. Article plus Supplemental Information [file mmc2.pdf]

Immunity

Article

Survival of Single Positive Thymocytes Depends upon Developmental Control of RIPK1 Kinase Signaling by the IKK Complex Independent of NF-κB

Graphical Abstract

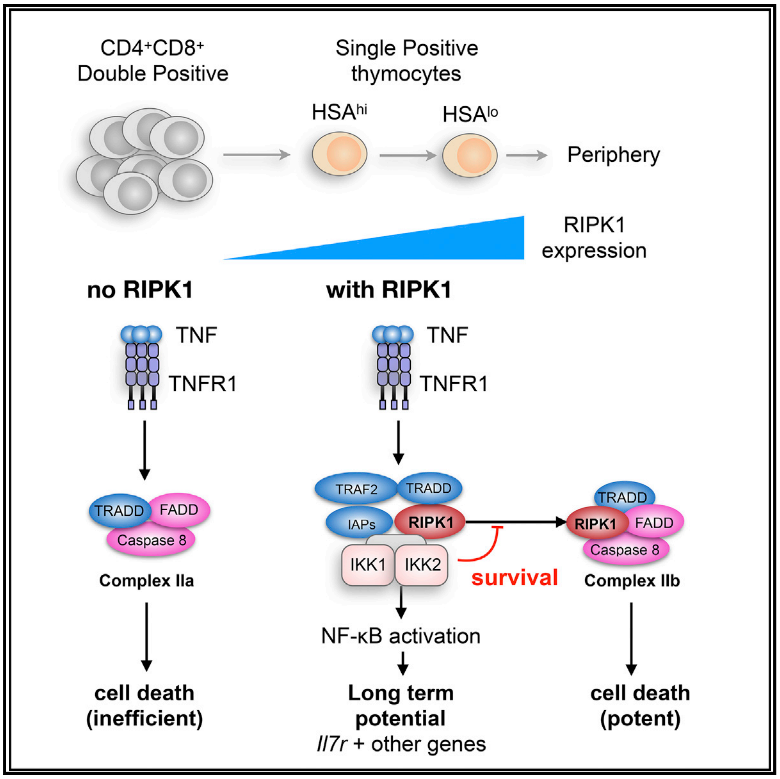

Highlights

- The IKK complex is essential for survival of SP thymocytes
- Developmental progression of SP thymocytes does not require canonical NF-κB
- Expression RIPK1 is induced in thymocytes following positive selection
- Survival of SP thymocytes depends upon active repression of RIPK1 by IKK

Authors

Louise V. Webb, Alessandro Barbarulo, Jelle Huysentruyt, ..., Steven Ley, Peter Vandenabeele, Benedict Seddon

Correspondence

benedict.seddon@ucl.ac.uk

In Brief

Survival of developing thymocytes depends upon activation of the IKK complex downstream of TNFR1 signaling by TNF. Webb and colleagues demonstrate that the pro-survival activity of IKK is mediated not by NF-κB activation but by direct repression of RIPK1 that otherwise triggers cell death in the absence of IKK activity.

Webb et al., 2019, Immunity *50*, 348–361
February 19, 2019 Crown Copyright © 2019 Published by Elsevier Inc.
https://doi.org/10.1016/j.immuni.2019.01.004

Cell Press

Immunity
Article

Survival of Single Positive Thymocytes Depends upon Developmental Control of RIPK1 Kinase Signaling by the IKK Complex Independent of NF-κB

Louise V. Webb,[2,6] Alessandro Barbarulo,[1,6] Jelle Huysentruyt,[3,4] Tom Vanden Berghe,[3,4] Nozomi Takahashi,[3,4] Steven Ley,[5] Peter Vandenabeele,[3,4] and Benedict Seddon[1,7,*]
[1]Division of Infection and Immunity, UCL Institute of Immunity and Transplantation, Royal Free Hospital, Rowland Hill Street, London NW3 2PF, UK
[2]Present address: Francis Crick Institute, Mill Hill Laboratories, London NW7 1AA, UK
[3]VIB-UGent Center for Inflammation Research, VIB-UGent Research Building FSVM, Technologiepark 927, Ghent 9052, Belgium
[4]Department of Biomedical Molecular Biology, Ghent University, Ghent, Belgium
[5]Division of Molecular Immunology, Imperial College, London, UK
[6]These authors contributed equally
[7]Lead Contact
*Correspondence: benedict.seddon@ucl.ac.uk
https://doi.org/10.1016/j.immuni.2019.01.004

SUMMARY

NF-κB (nuclear factor κB) signaling is considered critical for single positive (SP) thymocyte development because loss of upstream activators of NF-κB, such as the IKK complex, arrests their development. We found that the compound ablation of RelA, cRel, and p50, required for canonical NF-κB transcription, had no impact upon thymocyte development. While IKK-deficient thymocytes were acutely sensitive to tumor necrosis factor (TNF)-induced cell death, Rel-deficient cells remained resistant, calling into question the importance of NF-κB as the IKK target required for thymocyte survival. Instead, we found that IKK controlled thymocyte survival by repressing cell-death-inducing activity of the serine/threonine kinase RIPK1. We observed that RIPK1 expression was induced during development of SP thymocytes and that IKK was required to prevent RIPK1-kinase-dependent death of SPs *in vivo*. Finally, we showed that IKK was required to protect Rel-deficient thymocytes from RIPK1-dependent cell death, underscoring the NF-κB-independent function of IKK during thymic development.

INTRODUCTION

T lymphocytes develop in the thymus from pluripotent bone-marrow-derived progenitors through an ordered sequence of developmental events. There has been considerable interest in identifying those signaling pathways and transcriptional networks responsible for controlling different aspects of thymic development. The nuclear factor κB (NF-κB) family of transcription factors is implicated in the function and development of many tissues and cells (Bonizzi and Karin, 2004). Canonical NF-κB signaling is mediated by heterodimers or homodimers of p50, RelA, and cRel family members that are sequestered in the cytoplasm by inhibitory proteins, the inhibitors of kappa B (IκB) family, and related protein p105. Release of canonical NF-κB dimers is controlled by the inhibitor of kappa B kinase (IKK) complex, a trimeric complex of two kinases, IKK1 (IKKα) and IKK2 (IKKβ), and a third regulatory component, NEMO (IKKγ). IKK, activated by the upstream TAK1-TAB1 (transforming growth factor [TGF]-β-activated kinase 1 and TAK1-binding protein 1) kinase complex, phosphorylates IκB, resulting in its degradation by the proteosome and permitting NF-κB dimers to enter the nucleus. Previous studies suggest a role for NF-κB downstream of the preTCR (T cell receptor) complex in double negative (DN) thymocytes (Voll et al., 2000) and downstream of the TCR for the selection of CD8 (cluster of differentiation 8) lineage cells from DP (double positive) thymocytes (Hettmann and Leiden, 2000; Mora et al., 1999; Jimi et al., 2008). However, it is notable that TCR activation of the IKK complex is mediated via a signalosome complex comprising Card11, Bcl10, and Malt1 proteins (the CBM complex) that is not required for thymocyte development (Schmidt-Supprian et al., 2004).

More recent studies have instead suggested that NF-κB activation downstream of tumor necrosis factor (TNF) superfamily receptors (Tnfrsf), including TNF receptors (TNFR), is required during thymopoiesis specifically for development of single positive (SP) thymocytes. TNF stimulation results in a complex cascade of signaling events that can result in either cell death or survival (reviewed in Annibaldi and Meier, 2018; Vandenabeele et al., 2010a). Ligation of TNFR1 causes recruitment of TRADD (TNFR1-associated death domain protein), TRAF2, and the serine/threonine kinase RIPK1. The ubiquitin ligases TRAF2, cellular inhibitor of apoptosis proteins (cIAP), and the linear ubiquitin chain assembly complex (LUBAC) add ubiquitin chain modifications to themselves and to RIPK1, which creates a scaffold allowing recruitment and activation of the TAK1-TAB1 and IKK complexes and formation of the so-called TNFR complex I. Triggering IKK activity in this complex results in

 Crown Copyright © 2019 Published by Elsevier Inc.
This is an open access article under the CC BY license (http://creativecommons.org/licenses/by/4.0/).

activation of downstream NF-κB and promotion of cell survival. A failure to maintain the stability of complex I results in the formation of one of several cell-death-inducing complexes. Complex IIa is composed of TRADD, FADD (Fas-associated protein with death domain), and caspase-8 (Micheau and Tschopp, 2003; Wang et al., 2008), whereas in the presence of inhibitor of apoptosis protein (IAP) inhibitors, IKK inhibitors, or TAK1 inhibitors (Dondelinger et al., 2013, 2015; Legarda-Addison et al., 2009), additional recruitment of RIPK1 allows formation of related complex IIb. Both of these complexes induce apoptosis; in the former case it is RIPK1-kinase-activity independent, whereas in the latter case it is RIPK1-kinase dependent (Annibaldi and Meier, 2018; Dondelinger et al., 2016; Ting and Bertrand, 2016). In the absence of caspase-8 protease activity, RIPK3 and MLKL (mixed lineage kinase domain like pseudokinase) can be recruited to complex IIb and together trigger a distinct form of cell death termed necroptosis (for review see Pasparakis and Vandenabeele, 2015; Vandenabeele et al., 2010b).

The evidence that NF-κB signaling is required during SP development largely comes from disruptions to complex I formation. Ablation of the IKK complex, either by deletion of NEMO (Schmidt-Supprian et al., 2003) or by combined loss of IKK1 and IKK2 subunits (Webb et al., 2016), results in a developmental arrest in SP thymocytes at the immature HSA^{hi} stage. Similarly, an absence of TAK1 and LUBAC results in similar blocks in SP thymocyte development (Liu et al., 2006; Teh et al., 2016; Wan et al., 2006). TNF is implicated as a key trigger of these pathways because its blockade *in vivo* almost completely rescues SP development in IKK-deficient thymocytes (Webb et al., 2016) and rescues survival of TAK1-deficient thymocytes (Xing et al., 2016). Together, these studies suggest that TAK1- and IKK-dependent activation of NF-κB by TNF is required for thymocyte survival. Acquisition of proliferative competence by SP thymocytes is also suggested to require NF-κB signaling because TAK1-deficient thymocytes do not proliferate in response to TCR triggering, a defect rescued by expression of a constitutively active IKK2 transgene (Xing et al., 2016). Although these studies find clear NF-κB gene transcription profiles amongst SP thymocytes, it remains unclear which gene targets are functionally relevant for SP thymocyte development and survival or how cell death is controlled when complex I formation is compromised. One NF-κB gene target that has been functionally validated in thymocytes, however, is *Il7r* (Miller et al., 2014; Silva et al., 2014). Expression of interleukin-7 receptor (IL-7R) by newly developed T cells is triggered by signals from Tnfrsf members, including TNFR1 and CD27, and is dependent upon NF-κB signaling. Although *Il7r* gene induction is initiated in mature SP thymocytes, it is not required for SP development and only reaches maximal abundance in newly developed T cells after leaving the thymus. This induction of IL-7R expression is, however, essential for long-term survival of naive T cells (Silva et al., 2014).

NF-κB signaling has therefore been implicated in multiple developmental processes throughout thymopoiesis, but most specifically in post-selection thymocytes: (1) to protect thymocytes from cell death triggered by TNF, (2) for differentiation of SP thymocytes into functionally competent cells with migratory capacity, and (3) for homeostatic maturation of newly developed T cells, mediated in part by induction of IL-7R. In the present study, we sought to better understand how the IKK complex and NF-κB signaling downstream of TNF control SP thymocyte development and reveal RIPK1 as a central regulator of post-selection thymocyte death, survival, and maturation.

RESULTS

Development and Survival of SP Thymocytes Does Not Depend on NF-κB

To directly ask whether NF-κB signaling is required for SP thymocyte development, we generated mice with compound deficiencies of the three Rel family members required for canonical NF-κB signaling: RelA, cRel, and p50. *Cd4*^{Cre} *Rela*$^{fx/fx}$ (RelA$^{\Delta T}$) mice, *Nfkb1*$^{-/-}$ mice that lack p105 and p50, and *Rel*$^{-/-}$ mice that are cRel deficient were intercrossed, generating different combinations of Rel subunit deficiency. Thymic development of CD4 and CD8 SP thymocytes was largely normal in the different compound mutants, which had normal phenotypes and numbers of immature HSA^{hi}CD62L^{lo} and mature HSA^{lo}CD62L^{hi} SP thymocytes (Figures 1A and 1B). The exception was *Nfkb1*$^{-/-}$ thymocytes, which exhibited perturbed CD8-lineage development (Figure 1B), as previously described (Gugasyan et al., 2012). To confirm that canonical NF-κB signaling was absent in RelA$^{\Delta T}$ *Rel*$^{-/-}$ *Nfkb1*$^{-/-}$ triple-deficient thymocytes, we first tested their capacity to proliferate in response to CD3+CD28 stimulation. SP thymocytes from RelA$^{\Delta T}$ *Rel*$^{-/-}$ *Nfkb1*$^{-/-}$ donors were, as expected, unresponsive to CD3+CD28 cross-linking (Figure 1C). We then analyzed gene expression by RelA$^{\Delta T}$ *Rel*$^{-/-}$ *Nfkb1*$^{-/-}$ CD8 SP thymocytes. We have previously identified a number of NF-κB gene targets by transcriptional profiling of CD8 SP thymocytes from *Chuk*$^{fx/fx}$ *Ikbkb*$^{fx/fx}$ *huCD2*^{iCre} (IKKΔT^{CD2}) mice (Webb et al., 2016). Comparing gene expression between RelA$^{\Delta T}$ *Rel*$^{-/-}$ *Nfkb1*$^{-/-}$ and IKK-deficient CD8 SP thymocytes revealed a very similar pattern of gene regulation (Figure 1D). Previously defined targets such as IAPs, *Traf1* (TNF receptor associated factor 1), *Il7r*, *Bcl3* (B-cell lymphoma 3-encoded protein), *Tnfaip3* (TNF alpha induced protein 3, A20), and *Nfkbia* were all similarly reduced in both strains. Conversely, genes relevant to TNF signaling but not found to be regulated in IKK-deficient thymocytes, such as *Diablo*, *Cflar*, *Cyld*, and *Xiap*, were also unaffected in RelA$^{\Delta T}$ *Rel*$^{-/-}$ *Nfkb1*$^{-/-}$ cells. Additionally, we found that expression of both *Relb* and *Nfkb2*, associated with alternative NF-κB activation, appeared to be dependent on canonical NF-κB signaling because their mRNA abundance was substantially reduced in both IKK-deficient and RelA$^{\Delta T}$ *Rel*$^{-/-}$ *Nfkb1*$^{-/-}$ deficient mice. So, although only canonical Rel components were genetically targeted in RelA$^{\Delta T}$ *Rel*$^{-/-}$ *Nfkb1*$^{-/-}$ mice, thymocytes, in fact, lacked expression of all five subunits. Finally, we asked whether Rel-subunit-deficient thymocytes were more sensitive to TNF-induced cell death than controls were. Previous studies have suggested that thymocyte survival is dependent upon NF-κB activation, specifically to protect cells from TNF-induced cell death (Webb et al., 2016; Xing et al., 2016). However, all thymic SP subsets in RelA$^{\Delta T}$ *Rel*$^{-/-}$ *Nfkb1*$^{-/-}$ hosts were resistant to TNF-induced cell death, even at supra-physiologic concentrations (Figure 1E), despite a complete inability to transmit canonical NF-κB signals.

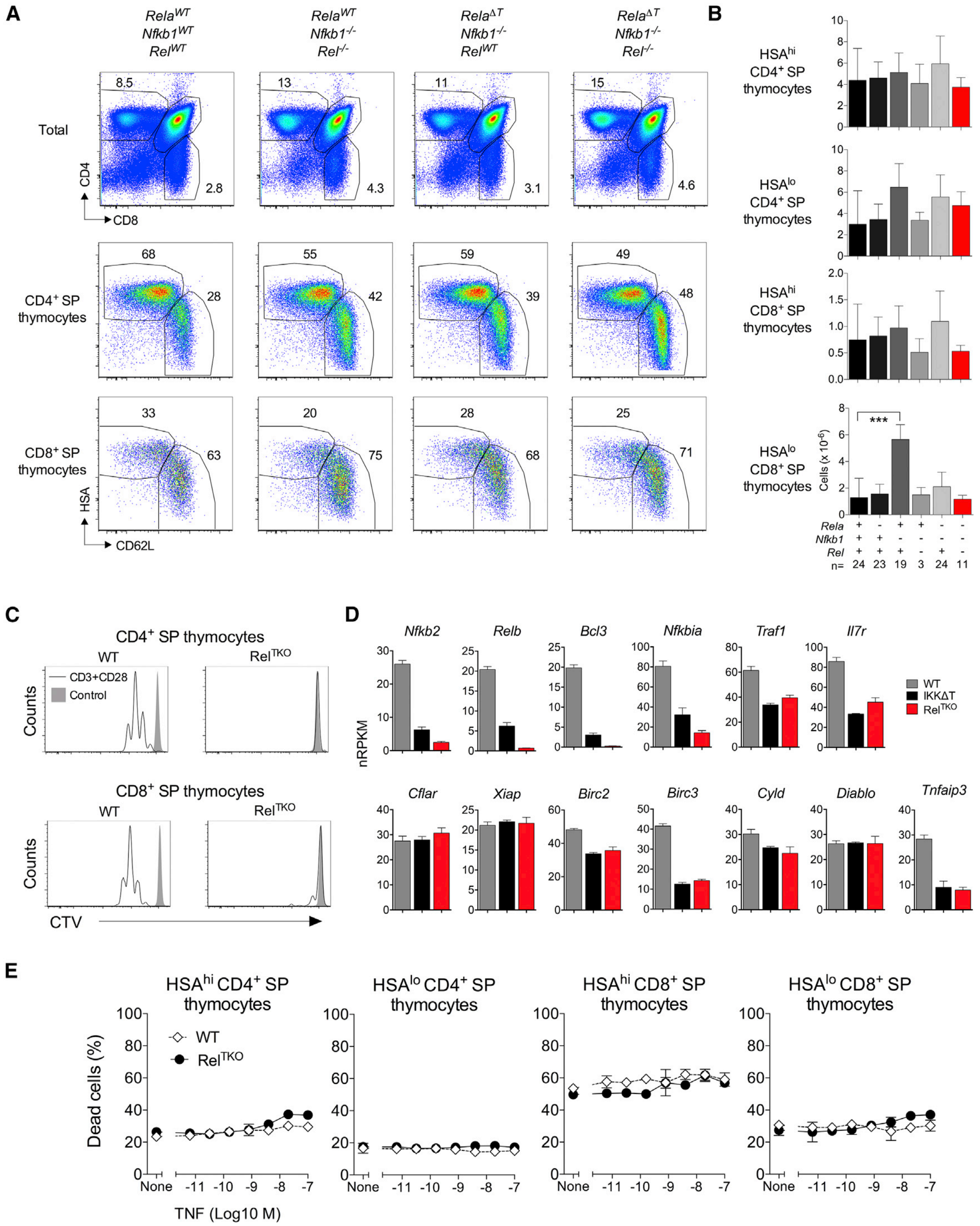

Figure 1. Normal Thymocyte Development and Resistance to TNF-Induced Cell Death in the Absence of Canonical NF-κB Signaling

(A) The phenotype of the indicated thymic populations (rows) from the indicated strains (columns), displayed as 2D plots of relative fluorescence of the indicated marker. See STAR Methods for electronic gating strategies.

(B) Total numbers of the indicated SP thymocyte subsets recovered from compound mutants of *Rela*, *Nfkb1*, and *Rel*, as indicated in the axis labels. +, WT, – , deficient. Numbers of mice (n) per group are indicated in first bar chart.

(C) CTV (cell trace violet) dilution by CD4^{+} and CD8^{+} SP thymocytes from the indicated strains stimulated as indicated for 72 h.

(D) Gene expression (normalized reads per kb exons per million reads, nRPKM) by CD8^{+} SP thymocytes from WT (n = 8) and RelA$^{\Delta T}$ *Nfkb1*$^{-/-}$ *Rel*$^{-/-}$ mice (Rel^{TKO}) (n = 5) was determined by RNA-seq analysis. Previously published RNA-seq of *Tnfrsf1a*$^{-/-}$ IKKΔT^{CD4} TCR^{hi} CD8^{+} SP thymocytes (n = 4; Webb et al., 2016) was included in the analysis.

(E) Total thymocytes from RelA$^{\Delta T}$ *Nfkb1*$^{-/-}$ *Rel*$^{-/-}$ and WT controls were cultured for 24 h with different doses of TNF. Graphs show percentage of dead cells. Data are pooled from six independent experiments (A and B) or are representative of three independent experiments (C and E). Error bars indicate SD.

Homeostatic Maturation of Newly Developed T Cells Is NF-κB Dependent

Because NF-κB was not required for thymocyte survival in response to TNF, we wanted to confirm whether NF-κB was indeed responsible for homeostatic maturation of T cells, as suggested by previous studies (Miller et al., 2014; Silva et al., 2014; Webb et al., 2016). To assess homeostatic maturation of T cells, we analyzed both total numbers of naive peripheral T cells and IL-7R expression in different Rel-subunit-deficient strains because *Il7r* is an NF-κB target gene in SP thymocytes and peripheral T cells (Miller et al., 2014; Silva et al., 2014). Mice lacking only RelA, only p105, or both p105 and cRel all had normal naive T cell numbers, although there was evidence of a modest reduction in IL-7R expression (Figure 2A). However, both naive T cell numbers and IL-7R expression were substantially reduced in mice lacking both p105 and RelA, whereas combined RelA, cRel, and p105 deficiency resulted in the most profound loss of naive T cells and IL-7R expression. Importantly, the extent to which naive T cell numbers and IL-7R abundance was reduced in RelA$^{\Delta T}$ *Rel*$^{-/-}$ *Nfkb1*$^{-/-}$ hosts closely resembled that of *Tnfrsf1a*$^{-/-}$ *Chuk*$^{fx/fx}$ *Ikbkb*$^{fx/fx}$ *Cd4*^{Cre} (*Tnfrsf1a*$^{-/-}$ IKKΔT^{CD4}) mice. Together, these data confirm that NF-κB is essential for homeostatic maturation of newly developed T cells, permitting maximal induction of IL-7R and survival of naive T cells. These experiments also provided evidence of Rel-subunit specificity in this process because RelA alone was sufficient for generation of normal naive T cell numbers, whereas cRel was not.

Finally, we assessed functional differentiation of SP thymocytes and T cells in Rel-deficient mice because acquisition of proliferative capacity by developing thymocytes is thought to be NF-κB dependent (Xing et al., 2016). We first examined memory and regulatory T (Treg) cell populations. Thymic development of Treg cells and generation of peripheral memory CD4^{+} T cells are both highly reliant upon cRel (Isomura et al., 2009; Zheng et al., 2003). Analysis of *Rel*$^{-/-}$ *Nfkb1*$^{-/-}$ mice confirmed the findings of these earlier studies but also revealed that although both populations were greatly reduced, they were not completely absent. In contrast, RelA$^{\Delta T}$ *Rel*$^{-/-}$ *Nfkb1*$^{-/-}$ mice were almost completely devoid of both Treg and CD4^{+} memory T cells (Figures 2B and 2C). Thymocytes from RelA$^{\Delta T}$ *Rel*$^{-/-}$ *Nfkb1*$^{-/-}$ mice failed to proliferate in response to TCR stimulation *in vitro* (Figure 1C). However, it was unclear whether the absence of CD4^{+} memory T cells in this strain was because naive T cells were functionally immature in the absence of NF-κB signaling in the thymus or because unresponsiveness simply reflected a non-redundant requirement for NF-κB signaling downstream of TCR in order to activate T cells and generate memory T cells. To address this question further, we assessed proliferative responses of thymocytes and peripheral T cells from IKK1-deficient donors in the presence of an IKK2 inhibitor. IKK1 deficiency alone has little impact upon thymocyte development (Chen et al., 2015), and so the impact of complete IKK blockade in mature T cells can be assessed. Responses to CD3 cross-linking by both thymocytes and peripheral T cells from IKK1-deficient mice was largely normal (Figure 2D). Blocking IKK2 in wild-type (WT) thymocytes had a modest effect on proliferation, whereas in IKK1-deficient thymocytes, additional blockade of IKK2 resulted in a profound inhibition of cell division, as expected (Figure 2D). However, a similar block in proliferation was also observed when otherwise responsive peripheral T cells from IKK1-deficient mice were activated in the presence of an IKK2 inhibitor. Therefore, the failure of IKK-deficient and Rel-deficient thymocytes and peripheral T cells to proliferate in response to TCR triggering most likely reflects an absolute requirement for this signaling pathway downstream of TCR triggering rather than a developmental defect.

Gene Dose of *Chuk* and *Ikbkb* Reveals Distinct Functions for the IKK Complex in Thymocyte Survival and Maturation

The finding that NF-κB was not required for thymocyte survival appeared contrary to earlier studies showing the critical requirement for the IKK complex during SP thymocyte development. We therefore examined the role of the IKK complex in more detail to better understand how both thymocyte survival and homeostatic maturation are regulated. First, we examined the function of individual IKK subunits and specifically whether there was a distinct reliance upon these subunits for thymocyte survival versus NF-κB-dependent induction of IL-7R. We examined T cell development in mice with a T-cell-specific deletion of *Chuk* or *Ikbkb* genes and in compound mutants expressing only single copies of either gene. Analyzing SP thymocyte compartments of mice lacking both *Chuk* and *Ikbkb* (IKKΔT) (Figure 3A) confirmed results of earlier studies reporting a profound loss of mature HSA^{lo} CD4^{+} and CD8^{+} SP T cell subsets in mice lacking IKK function (Schmidt-Supprian et al., 2003; Webb et al., 2016). Both IKK1 and IKK2 alone were sufficient for normal thymocyte survival because numbers of SP subsets in mice lacking either of the IKK subunits were normal (Figure 3A). There was some evidence that IKK2 was more potent than IKK1 at promoting survival. A single *Ikbkb* gene was sufficient for normal thymocyte survival in the absence of IKK1, whereas, in contrast, a single copy of the *Chuk* gene was sufficient for CD4^{+}, but not CD8^{+}, SP thymocyte subsets in the absence of IKK2.

To assess the impact of compound *Chuk* and *Ikbkb* subunit deletion upon homeostatic maturation of thymocytes, we measured the expression of IL-7R in peripheral T cells and the total number of peripheral naive T cells. Both induction of IL-7R expression (Figure 3B) and total naive T cell numbers (Figure 3C) were highly sensitive to compound deletions of *Chuk* and *Ikbkb* alleles, with clear evidence of gene dose effect. While thymocyte survival was only affected by the complete loss of both *Chuk* and *Ikbkb*, normal IL-7R expression and maximal naive T cell numbers required optimal expression of both IKK1 and IKK2. Together, these data reveal that survival of thymocytes and induction of IL-7R have distinct requirements for IKK activity, implying that IKK mediates these functions by distinct mechanisms.

RIPK1 Expression Is Developmentally Regulated in SP Thymocytes

Given the evidence that IKK may regulate survival and maturation of SP thymocyte subsets by distinct mechanisms, we wished to better understand how TNF signaling was controlled during thymopoiesis. We performed a meta-analysis of published transcriptomics data generated by RNA sequencing (RNA-seq; Sinclair et al., 2015) to examine the TNF signaling

CellPress

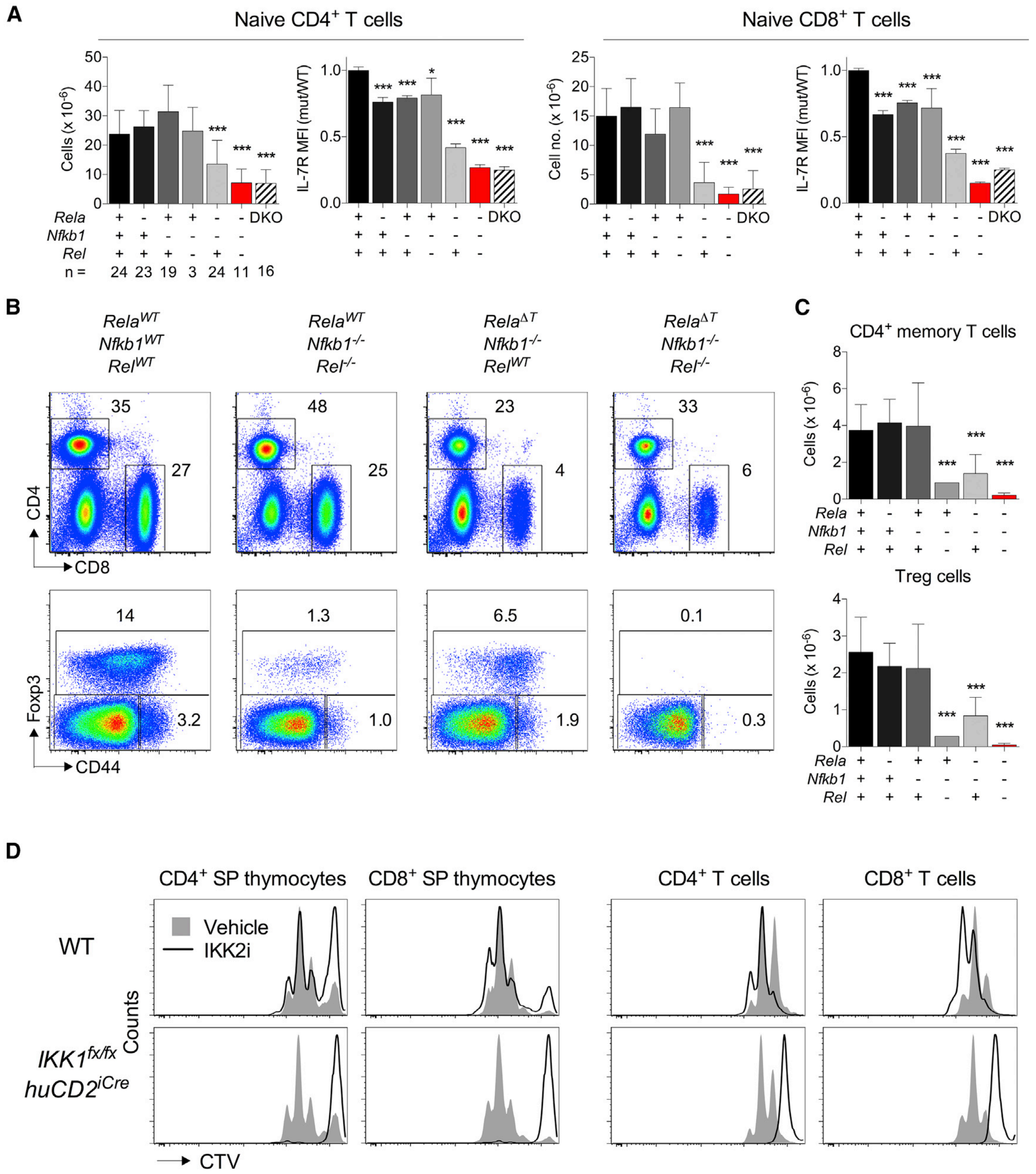

Figure 2. Homeostatic Maturation of T Cells Is NF-κB Dependent

(A) Total numbers of naive CD4+ and CD8+ T cells and their expression of IL-7R, from lymph node and spleen of the indicated strains (+, WT, –, deficient for specified locus). DKO (double knockout) - *Chuk*$^{fx/fx}$ *Ikbkb*$^{fx/fx}$ *Cd4*^{cre} *Tnfrsf1*$^{-/-}$ strain as control. Numbers of mice (n) analyzed per group are indicated in the x axis.

(B) Phenotype of total live lymph node cells and CD4+ T cells from the indicated strains, displayed as 2D plots of relative fluorescence of the indicated markers.

(C) Numbers of CD4+ memory T and Treg cells from the indicated strains.

(D) Sorted thymic populations from the indicated strains and total lymph node cells from the same mice were labelled with CTV and stimulated with CD3+CD28 mAb (monoclonal antibody) for 72 h in the presence of IKK2 inhibitor (IKK2i) or vehicle control. Histograms show relative fluorescence of CTV by different subsets. Data are the pool of six independent experiments (A–C) or are representative of three independent experiments. Error bars indicate SD. Significant differences versus WT are indicated in (A) and (C).

network in subpopulations of developing thymocytes, with particular attention upon developmental changes in mature SP thymocytes, because it is in these populations that TNF signaling appears to have the most impact. TNF signaling activates a number of pathways via a complex cascade of intracellular adaptors, kinases, and ubiquitinating enzymes (Figure 4A). Analyzing gene expression in different DP and SP thymocyte subsets revealed that expression of many of the signaling intermediaries was

unchanged as thymocytes underwent differentiation. There were notable exceptions to this, though, that included *Ripk1* and *Casp8*, which were up-regulated in total CD8$^+$ SPs and HSA^{lo} CD4$^+$ SPs, but not in immature HSA^{hi}CD4$^+$ SPs or DP thymocyte subsets. This pattern of expression correlated well with the susceptibility of different subsets to TNF-induced cell death, apparent in the absence of IKK expression. RIPK1 is a serine/threonine kinase critical for induction of TNF-induced cell death and is a major survival factor through its scaffold function involving both NF-κB-dependent and -independent mechanisms (Pasparakis and Vandenabeele, 2015). Similarly, caspase-8 is a target of RIPK1 kinase activity required for induction of apoptosis by TNF (Dondelinger et al., 2016; Ting and Bertrand, 2016; Vandenabeele et al., 2010a). To confirm the developmental pattern of *Ripk1* expression suggested by RNA-seq, we measured RIPK1 protein abundance at the single-cell level by flow cytometry (Figure S1). RIPK1 protein was undetectable amongst the most immature DP thymocyte subsets (DP1 and DP2), but expression was induced progressively throughout SP development, reaching maximum abundance in HSA^{lo} SP subsets (Figure 4B). Because IKK-deficient thymocytes are specifically susceptible to TNF-induced cell death (Webb et al., 2016), we also confirmed that the same pattern of RIPK1 expression was observed in thymocytes from *Tnfrsf1a*$^{-/-}$ IKKΔT^{CD4} mice (Figure 4B) and that RIPK1 was not itself regulated by NF-κB signaling. The close correlation between RIPK1 abundance and TNF reactivity of thymic subsets suggested that RIPK1 could be a central regulator of thymocyte survival.

Normal Thymopoiesis, but Reduced Peripheral T Cell Numbers in the Absence of RIPK1

To better understand the role RIPK1 plays in thymocyte survival and differentiation, we first analyzed T cell development in the absence of RIPK1. RIPK1 deficiency is associated with perinatal lethality (Kelliher et al., 1998). Therefore, we examined mice in which *Ripk1* expression is specifically ablated in T cells, using a conditional *Ripk1*$^{fx/fx}$ *Cd4*^{cre} strain. Analyzing the thymic phenotype revealed largely normal development in the absence of RIPK1 expression (Figure 5A), though there was some evidence of a modest reduction in mature HSA^{lo}CD62L^{hi} CD8$^+$ SP thymocytes in younger mice (Figure 5B). In contrast, the periphery of the *Ripk1*$^{fx/fx}$ *Cd4*^{cre} mice was profoundly T cell deficient, with substantial reductions in numbers of naive CD4$^+$ and CD8$^+$ T cells in both younger and older mice (Figure 5C). Since RIPK1 is thought to be required for optimal triggering of NF-κB signaling, it was possible that a failure to induce normal IL-7R expression could contribute to the reduction in peripheral T cells. Upregulation of IL-7R by newly developed T cells as they leave the thymus is NF-κB dependent (Silva et al., 2014), whereas initial induction

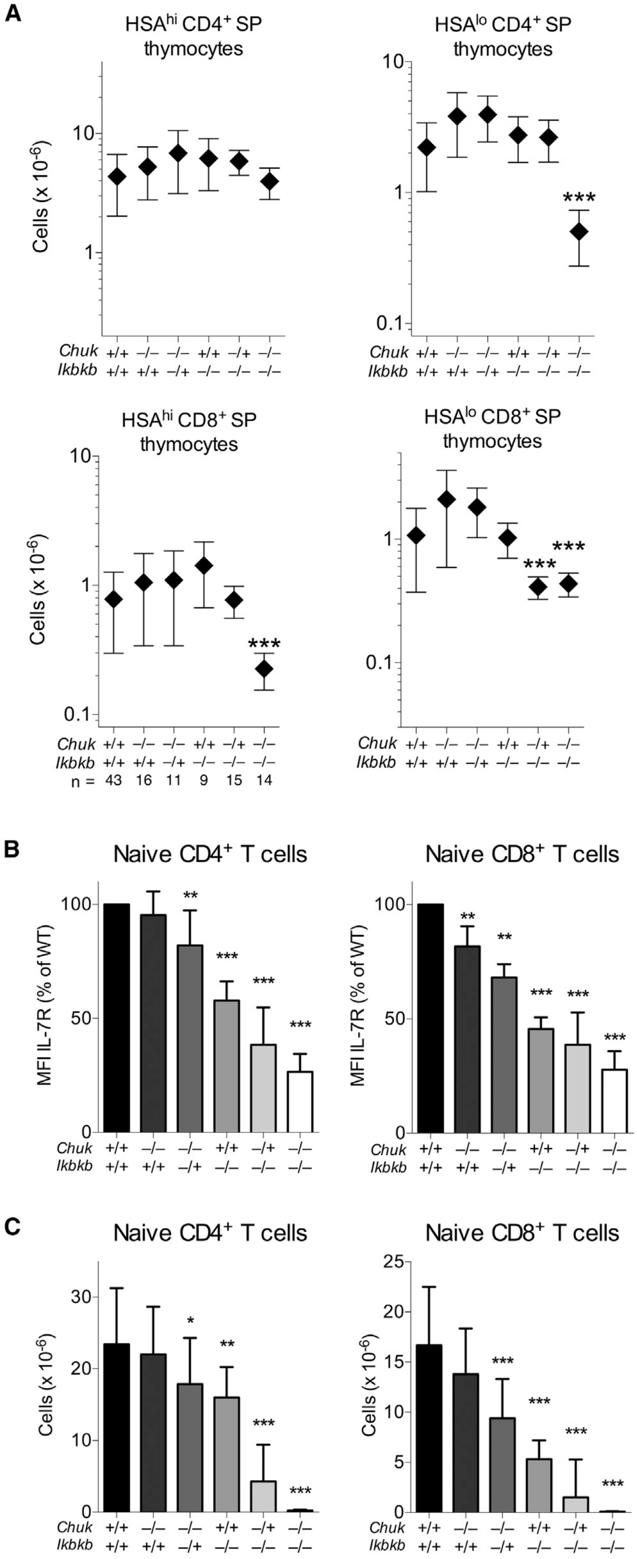

Figure 3. Distinct Requirements for IKK1 and IKK2 Expression for Thymocyte Survival and Peripheral T Cell Homeostasis

(A) Total numbers of thymocyte subsets from mice with combinations of *Chuk* and *Ikbkb* gene deletions indicated in the axis label (+ indicates WT allele, – indicates floxed allele, all mice cre+). Numbers of mice (n) analyzed per group are indicated in the x axis.

(B) Charts show IL-7R MFI (mean fluorescence intensity) of the indicated genotype by CD4$^+$ and CD8$^+$ naive T cells.

(C) Total numbers of CD4$^+$ (left) and CD8$^+$ (right) naive T cells recovered from combined lymph node and spleen of mice with the indicated combinations of *Chuk* and *Ikbkb* deletion.

Data are the pool of eight independent experiments with 3–18 mice per genotype. Error bars indicate SD. Significant differences versus WT are indicated.

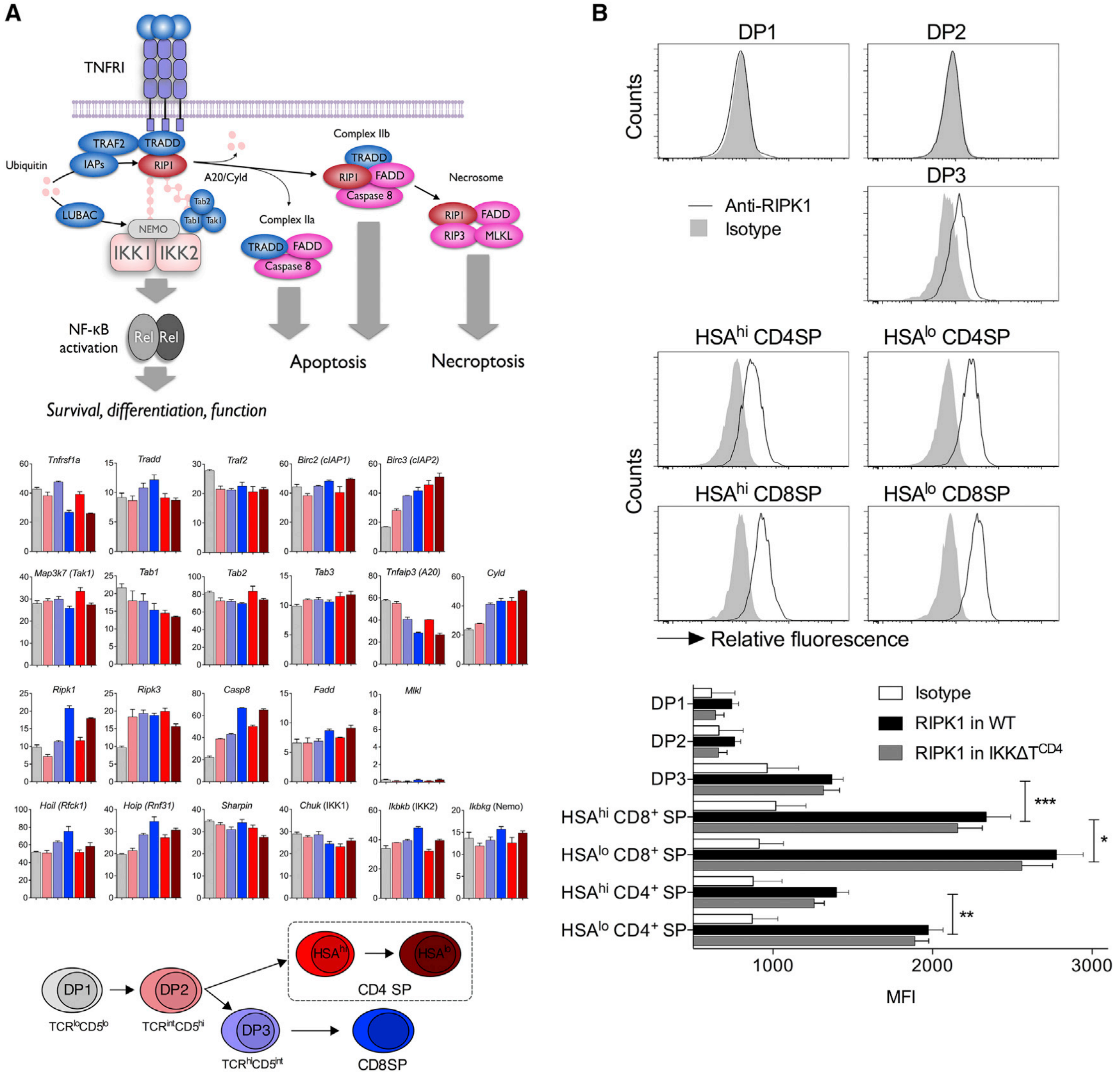

Figure 4. RIPK1 Expression Is under Developmental Regulation during Thymopoeisis

(A) Meta-analysis of RNA-seq data (Sinclair et al., 2015). The indicated thymocyte subsets were sorted from WT (n = 3) mice, mRNA was purified, and gene expression was determined by RNA-seq analysis. Bar charts show mRNA expression (nRPKM) of the indicated genes.

(B) Histograms are of anti-RIPK1 staining of the indicated thymocyte subpopulation as compared with isotope staining control. Bar charts are of MFI of anti-RIPK1 staining or isotype control of the indicated subsets from either WT (n = 4) or *Tnfr1*$^{-/-}$ IKKΔT^{CD4} mice (n = 2).

Data are representative of four independent experiments. Error bars indicate SD. Significant differences in RIPK1 expression between subsets were tested on pool of WT and *Tnfr1*$^{-/-}$IKKΔT^{CD4} data. Please also see Figure S1.

following thymocyte selection depends upon signaling of TCR but not of NF-κB (Silva et al., 2014; Sinclair et al., 2011). Analyzing IL-7R induction during thymic development of RIPK1-deficient T cells revealed normal induction following positive selection but a failure to up-regulate expression in the periphery (Figure 5D). Together, these data suggest that RIPK1 expression is required for peripheral T cell survival and optimal NF-κB-dependent induction of IL-7R.

RIPK1 Both Protects and Sensitizes Thymocytes to TNF-Induced Cell Death

RIPK1 is most highly expressed by those SP populations that are dependent upon IKK activity for survival. We therefore asked whether RIPK1 was required to regulate TNF-induced cell death in developing thymocytes. First, we examined survival of RIPK1-deficient thymocytes after TNF stimulation. In WT mice, DP and HSA^{hi} CD4^{+} SP thymocytes are modestly sensitive to TNF-induced cell death, whereas other more mature subsets are resistant (Webb et al., 2016) (Figure 6A). In contrast, RIPK1 deficiency rendered all thymic subsets sensitive to TNF-induced cell death to the same modest extent observed in DP and HSA^{hi} CD4^{+} SP thymocytes (Figure 6A). Therefore, the resistance of HSA^{lo} CD4 SP and CD8^{+} SP thymocyte subsets to TNF-induced death was RIPK1 dependent (Figure 6A), and the weak susceptibility of DP and HSA^{hi} CD4^{+} SP thymocytes in WT mice likely reflected the low or absent expression of

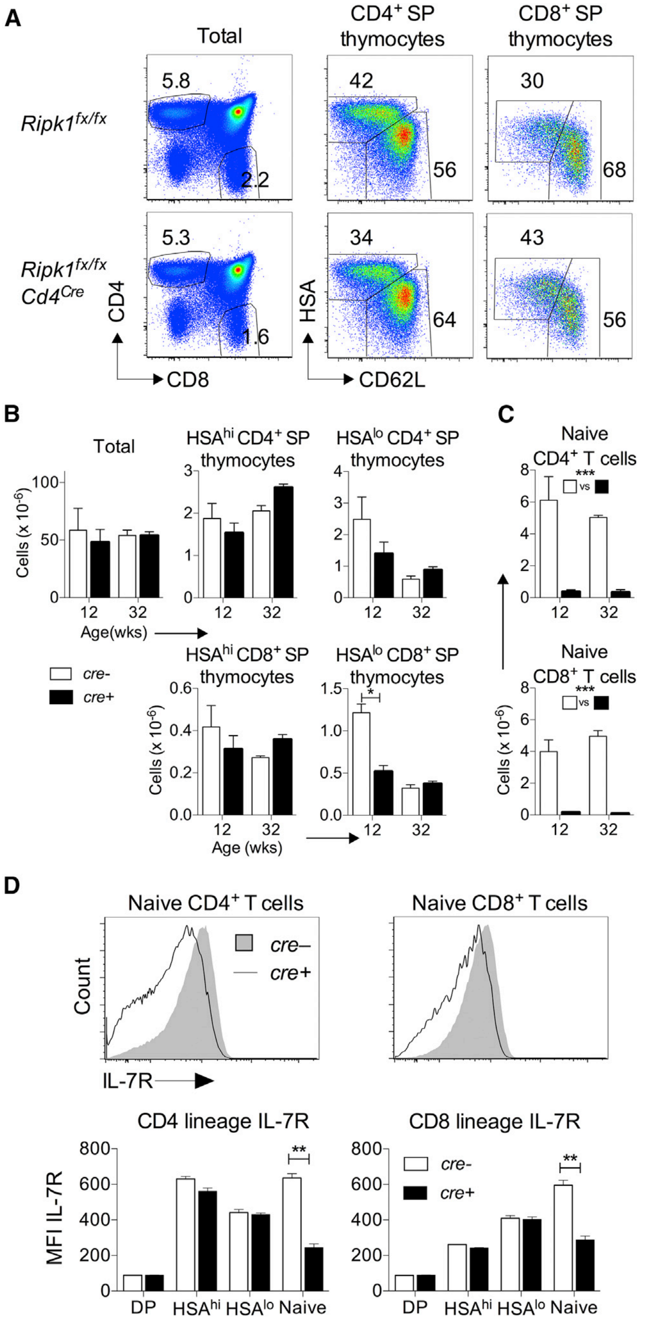

Figure 5. Normal Thymocyte Development, but Defective Peripheral Homeostasis in the Absence of RIPK1 Expression

Ripk1$^{fx/fx}$ *Cd4*^{cre} and *cre*$^{-}$ littermates were analyzed at 12 and 32 weeks of age.

(A) Phenotype of the indicated thymocyte populations (columns) from *cre*$^{+}$ and *cre*$^{-}$ *Ripk1*$^{fx/fx}$ mice at 12 weeks of age, displayed as 2D plots of relative fluorescence of the indicated marker.

(B) Total cell numbers of thymocytes and the indicated thymic subsets recovered from *cre*$^{+}$ (n = 5) and *cre*$^{-}$ (n = 5) *Ripk1*$^{fx/fx}$ mice.

(C) Total numbers of CD4^{+} and CD8^{+} naive T cells recovered from spleens of *cre*$^{+}$ (n = 3–5) and *cre*$^{-}$ (n = 3–5) *Ripk1*$^{fx/fx}$ mice. ***p < 0.001 by two-way ANOVA.

(D) Histograms of relative fluorescence of IL-7R by the indicated lymph node T cell subsets from representative *cre*$^{+}$ and *cre*$^{-}$ *Ripk1*$^{fx/fx}$ mice. Average IL-7R expression by the indicated thymic DP and SP (HSA^{hi} and HSA^{lo}) subsets and peripheral naive populations of CD4^{+} and CD8^{+} lineage cells.

Data are the pool of two independent experiments. Error bars indicate SD.

RIPK1. Together, these data suggest that induction of RIPK1 expression during development renders thymocytes protected from TNF-induced cell death.

Both expression and kinase activity of the IKK complex are required to protect thymocytes from TNF-induced cell death (Webb et al., 2016). While RIPK1 expression appeared to protect mature thymocytes from TNF-induced death, RIPK1 kinase activity is also a potent trigger of cell death (Annibaldi and Meier, 2018; Dondelinger et al., 2016; Ting and Bertrand, 2016). We therefore tested if TNF-induced death was dependent on RIPK1 kinase activity in thymocytes. We tested this first by sensitizing IKK1-deficient thymocytes to TNF-induced cell death with an IKK2 inhibitor and asked if RIPK1 kinase inhibitor Necrostatin 1 (Nec1) could prevent cell death. Following IKK blockade, TNF-induced cell death of SP subsets was completely blocked by the Nec1 inhibitor, whereas death of DP thymocytes by TNF was not (Figure 6B). Both the maximal extent of cell death and the concentration of Nec1 required to prevent TNF-induced death varied between different subsets. Since expression of RIPK1 differed between thymic populations, it was possible that cells with a higher abundance of RIPK1 would be more likely to die, reflected in the maximal extent of death, and/or would require more Nec1 to block cell death if RIPK1 kinase activity was a rate-limiting parameter. We therefore calculated half maximal inhibitory concentrations (IC50s) of Nec1 for individual subsets (Figure 6C) and compared both maximal extents of cell death and IC50s with RIPK1 protein levels (Figure 6D). This revealed strong correlations between RIPK1 abundance and both maximal cell death (r2 = 0.78) and IC50 of Nec1 (r^2 = 0.63). Similarly, we asked whether RIPK1 abundance also predicted susceptibility to TNF-induced cell death. Correlating published lethal dose 50 (LD50) of TNF (Webb et al., 2016) with RIPK1 abundance revealed a strong inverse correlation (r^2 = 0.88) (Figure 6D). Populations with the highest abundance of RIPK1 expression required the least TNF to trigger cell death. Together, these data show that TNF-induced death of thymocytes *in vitro* in the absence of IKK activity is RIPK1 dependent and further suggests that RIPK1 is rate limiting for TNF-induced cell death amongst SP thymocytes.

IKK Activity Is Required to Prevent RIPK1-Induced Cell Death Independently of NF-κB Signaling

Our data suggest that RIPK1 is a central regulator of thymocyte survival and that IKK kinase activity is required to repress RIPK1-kinase-dependent cell death by a mechanism independent of NF-κB. Recent studies show that RIPK1 is a direct target of IKK kinase activity in embryonic fibroblasts and that phosphorylation of RIPK1 by IKK represses its kinase activity and subsequent induction of cell death (Dondelinger et al., 2015). However, the significance of this regulatory circuit *in vivo* is unclear, and the activity of such an IKK-RIPK1 pathway has not so far been identified in a physiological setting. To ask whether thymocyte survival utilizes an NF-κB-independent function of IKK involving repression on RIPK1 *in vivo*, we first tested whether the death of IKK-deficient thymocytes *in vivo* was dependent upon RIPK1 kinase activity. We generated IKK-deficient mice in which RIPK1 kinase activity was impaired, introducing the inactivating RIPK1^{D138N} mutation (Newton et al., 2014) to the IKKΔT^{huCD2} strain. In this way, the scaffolding function of RIPK1 is preserved

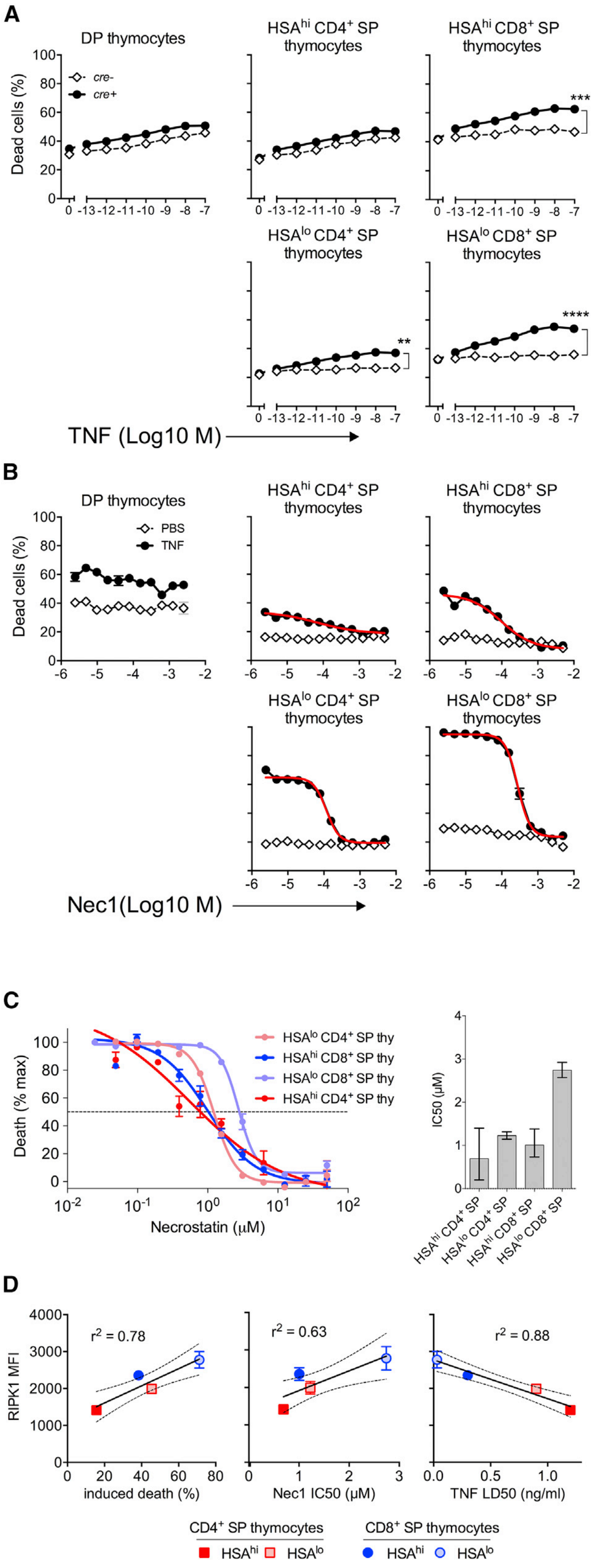

Figure 6. Induction of RIPK1 Expression Sensitizes Developing Thymocytes to TNF-Induced Cell Death

(A) Thymocytes from *cre*$^{+}$ and *cre*$^{-}$ *Ripk1*$^{fx/fx}$ mice were cultured with different doses of TNF for 24 h, and cell viability was determined by flow cytometry. Graphs show percentage of dead cells among the indicated thymic subpopulation from *cre*$^{+}$ and *cre*$^{-}$ *Ripk1*$^{fx/fx}$ mice (n = 3 each). Significance tested by one-way ANOVA.

(B and C) Total thymocytes from huCD2^{iCre} *Chuk*$^{fx/fx}$ mice were cultured with IKK2 inhibitor, TNF (10 ng/ml) or PBS, and different doses of RIPK1 kinase inhibitor Necrostatin-1 (Nec1). Line graphs show percentage of dead cells in cultures with TNF or PBS (B). Red lines (B) indicate modeled fits used to identify maximal death in different subsets and to estimate IC50 of Nec1 (C). Error bars of IC50 estimates indicate 95% confidence intervals.

(D) Scatter plots are of RIPK1 MFI from Figure 4B versus percentage of induced death (B), Nec1 IC50 estimated in (C), and TNF LD50 for IKK-deficient thymocytes (Webb et al., 2016). Solid lines are regression fits to data with 95% confidence indicated (dashed lines).

Data are representative of two (A) or three (B, C, and D) independent experiments.

while its kinase function is inactivated. In IKKΔT^{huCD2} mice, the expression of kinase-dead RIPK1^{D138N} resulted in a substantial rescue of SP development and export of T cells into the periphery. Development of mature HSA^{lo}CD62L^{hi} CD4^{+} SP and immature HSA^{hi}CD62L^{lo} CD8^{+} SP thymocytes was restored to near normal numbers in IKKΔT^{huCD2} RIPK1^{D138N} mice, while mature HSA^{lo}CD62L^{hi} CD8^{+} SP thymocytes were partially rescued (Figures 7A and 7B). Peripheral lymphoid organs of IKKΔT^{huCD2} mice were almost completely devoid of naive CD4^{+} or CD8^{+} T cells (Figures 7C and 7D). The few naive T cells present were mostly R26^{RYFP}-*cre*-reporter negative (Figure 7D) and therefore most likely represented rare deletion escapants that had failed to excise *Ikk* genes (Webb et al., 2016). In contrast, IKKΔT^{huCD2} RIPK1^{D138N} mice contained significant numbers of naive CD4^{+} and CD8^{+} T cells compared with IKKΔT^{huCD2} mice, which were R26^{RYFP} +ve, confirming that restoration of thymic development in these mice was also associated with export of IKK-deficient T cells. However, RIPK1^{D138N} did not restore peripheral T cell numbers in IKKΔT^{huCD2} mice to normal (Figure 7D), suggesting that IKK function was still required for sustained population of peripheral compartments.

We next asked whether RIPK1 is phosphorylated following TNF stimulation of thymocytes and, if so, if it was dependent upon IKK activity. We first confirmed that RIPK1 phosphorylation was detectable in WT thymocytes after immunoprecipitation of complex I. Indeed, deubiquitination and dephosphorylation of RIPK1 revealed characteristic electrophoretic shifts associated with ubiquitination and phosphorylation of RIPK1 within complex I after TNF stimulation (Figure 7E). Little phosphorylation of RIPK1 was detectable when WT thymocytes were preincubated with a pan-IKK inhibitor (Waelchli et al. 2006), whereas phosphorylation was unaffected by Nec1 (Figure 7F). In agreement with this, little phosphorylation of RIPK1 was detectable after TNF stimulation of IKK-deficient thymocytes from IKKΔT^{huCD2} RIPK1^{D138N} mice (Figure 7G), all together suggesting that RIPK1 phosphorylation in response to TNF stimulation is IKK dependent. Finally, we tested whether survival of RelA^{ΔT} *Rel*$^{-/-}$ *Nfkb1*$^{-/-}$ thymocytes in response to TNF was dependent upon IKK kinase activity, even though downstream NF-κB activation was completely absent. We measured survival of TNF-stimulated

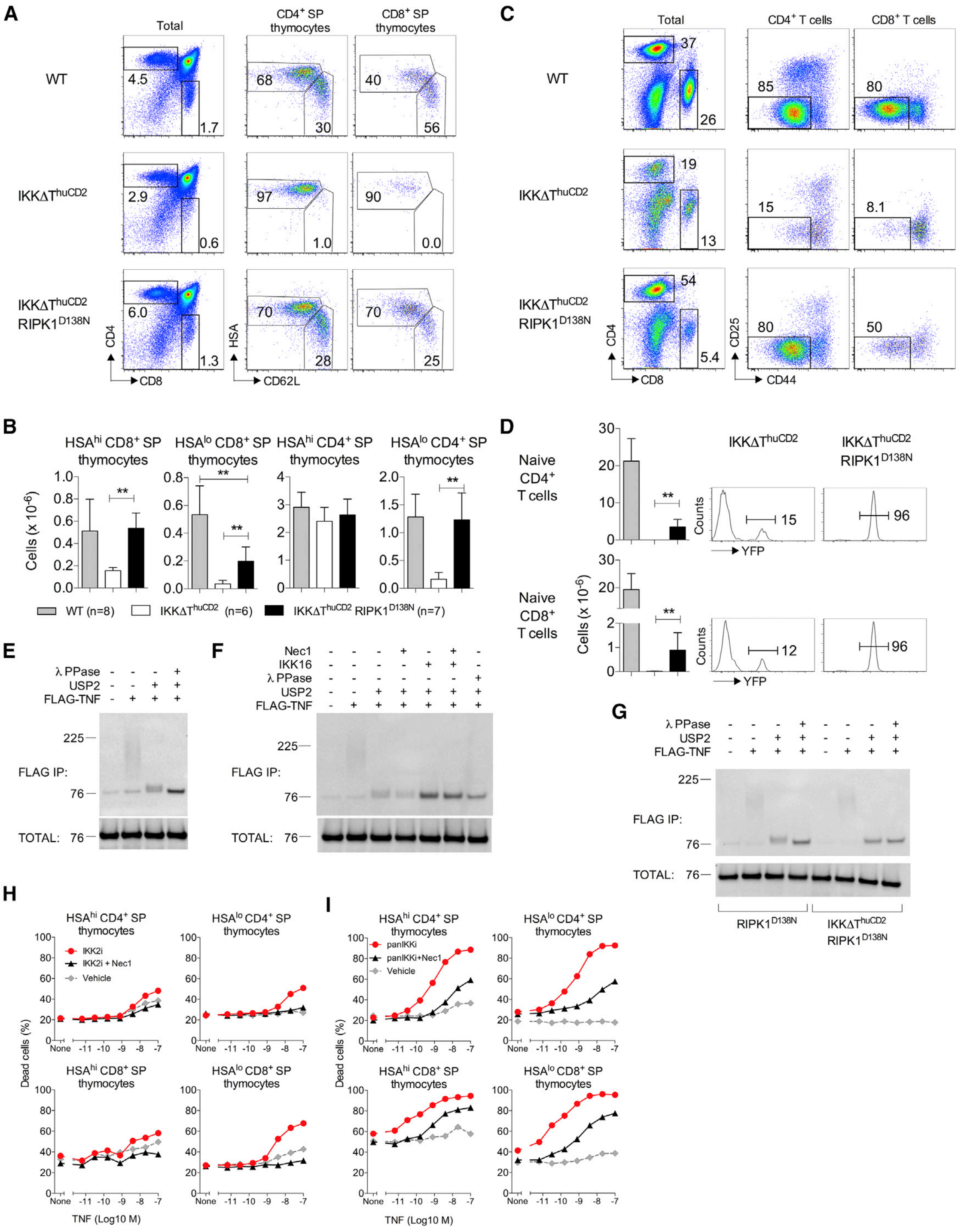

Figure 7. Kinase-Dead RIPK1^{D138N} Rescues Thymocyte Development in IKK-Deficient Hosts

(A–D) Phenotype, displayed as 2D plots of relative fluorescence, of thymus (A) and lymph nodes (C) and numbers of the indicated populations of thymocytes (B) and lymph nodes (D) from the indicated mouse strains.

(E–G) WT thymocytes (E and F) or thymocytes with the indicated genotype (G) were stimulated for 10 min with FLAG-TNF in the presence or absence of the indicated compounds, and FLAG immunoprecipitates were incubated with deubiquitylating (USP2) or phosphatase (λ PPase) enzymes as indicated. Extracts were analyzed by immunoblotting for RIPK1. Blots are representative of five (E), two (F), or four (G) independent experiments.

(H and I) Total thymocytes from RelA$^{\Delta T}$ *Nfkb1*$^{-/-}$ *Rel*$^{-/-}$ mice were cultured for 24 h with TNF and either IKK2 inhibitor (IKK2i) (H) or a pan IKK inhibitor (panIKKi) (I), and viability was determined by flow cytometry.

Data are representative (A, C, H, and I) or the pool (B and D) of four or more independent experiments. Error bars indicate SD.

thymocytes when IKK activity was blocked with two distinct IKK inhibitors: a specific IKK2 inhibitor, BI605906, that does not target IKK1 and therefore achieves a partial IKK inhibition (Clark et al., 2011) and a pan IKK inhibitor, IKK16 (Waelchli et al., 2006), that inhibits both IKK1 and IKK2. Specifically inhibiting IKK2 resulted in a clear sensitization of mature HSAlo SP thymocytes to TNF-induced cell death (Figure 7H), whereas culture with pan-IKK inhibitor rendered all SP populations sensitive to TNF-induced cell death (Figure 7I). To test whether cell death of RelA$^{\Delta T}$ *Rel*$^{-/-}$ *Nfkb1*$^{-/-}$ thymocytes following IKK inhibition was also RIPK1 dependent, cells were additionally cultured with Nec1. Importantly, TNF-induced cell death in the presence of IKK2 inhibition was completely blocked by Nec1, whereas death in cultures with IKK16 was substantially reduced by blocking RIPK1 kinase (Figures 7H and 7I), revealing that IKK does regulate RIPK1 kinase activity even in the absence of NF-κB. Taken together, our data suggest that IKK regulates thymocyte survival by repressing RIPK1 kinase activity by a mechanism that does not rely upon NF-κB activation.

DISCUSSION

Here, we found that mice lacking canonical NF-κB signaling in T cells undergo unperturbed thymic development and were resistant to TNF-induced cell death, even at supra-physiologic concentrations. Although it is clear that NF-κB activation is essential to control cell death processes in other tissues, such as hematopoietic progenitors and endothelial cells (Xu et al., 2018), this does not appear to be the case in thymocytes. Mice lacking upstream regulators of NF-κB activity, such as the IKK complex or TAK1, reveal a profound block in SP thymocyte development and were used to implicate NF-κB as an important downstream target mediating SP thymocyte development. We observed unperturbed SP development in RelA$^{\Delta T}$ *Rel*$^{-/-}$ *Nfkb1*$^{-/-}$ mice, with normal downregulation of HSA and induction of CD62L, thymic egress, and population of peripheral compartments. Transcriptomic analyses reveal a clear NF-κB-dependent gene signature in developing SP thymocytes (Webb et al., 2016; Xing et al., 2016), including genes that regulate cell survival, so it was natural to conclude that such genes should be responsible for resistance to TNF-induced cell death. However, our results clearly demonstrate that acute survival of thymocytes does not depend upon NF-κB regulated genes.

Instead, we identified a critical role for an IKK-RIPK1 signaling axis for controlling the survival of developing thymocytes. Our data suggest that IKK expression and kinase activity are essential to prevent TNF-induced RIPK1-mediated cell death *in vivo*. We showed that RIPK1 expression is induced during thymopoiesis reaching peak abundance in SP thymocytes after positive selection, and it is in these subsets that this pathway was most critical. In the absence of IKK function, induction of RIPK1 expression in HSA^{lo} CD4^{+} SP and CD8^{+} SP thymocytes coincided with extensive cell death of these specific subsets. Blocking RIPK1 kinase activity, both *in vitro* with inhibitors and *in vivo* in mice expressing kinase-dead RIPK1, rescued survival of these populations, revealing that repression of RIPK1-kinase-dependent cell death was the target of IKK kinase activity. However, we also found evidence, paradoxically, that RIPK1 expression may also have a protective function in thymocytes. In WT mice, DP thymocytes expressed little or no RIPK1 but were weakly sensitive to TNF-induced cell death. We confirmed that cell death was RIPK1 independent because RIPK1-deficient DP cells exhibited similar extents of cell death in response to TNF. What is more, RIPK1-deficient thymocytes maintained a similar, modest susceptibility to TNF-induced death throughout DP and SP development, in contrast to WT thymocytes that became resistant to TNF-induced cell death as RIPK1 expression was induced. This modest extent of TNF-induced death may reflect the TNFR complex IIa formation that occurs in the absence of RIPK1. Therefore, induction of RIPK1 expression may protect developing thymocytes from TNF-induced cell death by preventing complex IIa formation but also necessitates IKK activity to prevent the more potent activity of RIPK1-kinase-dependent, complex-IIb-mediated cell death in its place.

The most direct interpretation of our data is that IKK activity is required to repress RIPK1-kinase-dependent cell death independently of NF-κB. In support, studies in mouse embryonic fibroblasts reveal that the IKK complex can directly regulate RIPK1 during the assembly of the TNFR complex I. Phosphorylation of RIPK1 by IKK prevents its activation and consequently its integration into the death-inducing complex IIb (Dondelinger et al., 2015). The same study found that either IKK1 or IKK2 alone were sufficient for this regulatory function upon RIPK1. Consistent with this, survival of thymocytes lacking either IKK1 or IKK2 was normal, though we found evidence that IKK2 was a more potent regulator of RIPK1 because a single functional allele of *Ikbkb*, but not *Chuk*, was sufficient to protect thymocytes from TNF-induced death. Of significance, RIPK1 phosphorylation is also defective in the absence of cIAPs, TAK1, and NEMO (Dondelinger et al., 2015; Geng et al., 2017). Therefore, it seems that the block in SP thymocyte development observed in LUBAC- (Teh et al., 2016), TAK1-, and NEMO-deficient mice (Schmidt-Supprian et al., 2003; Teh et al., 2016; Xing et al., 2016) results from disruption of the delicate equilibrium of pro-death and pro-survival TNFR signaling complexes rather than from their common function of activating NF-κB. The potency of IKK2 in controlling RIPK1 may also be of relevance to the application of constitutively active IKK2 constructs, often used to reconstitute NF-κB signaling. It is possible that some functions of these constructs may instead be mediated by their direct targeting of RIPK1.

Our data suggest that acute survival of developing thymocytes is regulated by a direct IKK-RIPK1 pathway. If so, what function does NF-κB signaling then play during thymic development? Although it has been suggested that NF-κB signaling in developing SP thymocytes is critical for their acquisition of proliferative capacity to TCR stimulation (Xing et al., 2016), our results were equivocal on this point. Thymocytes were unable to proliferate in response to TCR triggering in the absence of either canonical NF-κB or IKK kinase activity. However, it was also clear that in normally peripheral T cells, blocking IKK kinase had the same impact upon proliferation, confirming that an intact IKK-NF-κB pathway is critical for normal responses to TCR triggering. Therefore, there is currently no evidence that the failure to trigger cell division in thymocytes with defective NF-κB activation represents anything other than an acute requirement for this pathway downstream of TCR stimulation. To test whether NF-κB activity in developing thymocytes is required for their

proliferative competence will require alternative approaches that can separate the acute requirement for NF-κB activation downstream of TCR from developmental programming.

Although NF-κB appears dispensable for normal thymocyte survival, our data strongly suggest that one function of NF-κB in newly developed T cells is instead to trigger a program of homeostatic maturation required for long-term survival of mature peripheral T cells. This function is clearly revealed in mice in which NF-κB signaling is absent and RIPK1-induced cell death is held in check. In such cases, thymocyte development and egress are largely normal, but peripheral T cells fail to survive long term and accumulate. Common to these strains is a failure of peripheral T cells to maintain high abundance of IL-7R, whose gene is a key NF-κB target required for their survival (Silva et al., 2014; Webb et al., 2016). However, numerous pro-survival genes, such as cIAPs, are targets of NF-κB signaling in the thymus (Webb et al., 2016), and although these have been originally thought to mediate acute survival of thymocytes, they might instead be more important for the long-term survival of peripheral T cells. Studies in which embryonic lethality of RIPK1 deficiency is rescued by elimination of caspase-8 or FADD in combination with either RIPK3 or MLKL (Dillon et al., 2014; Rickard et al., 2014) also suggest that thymopoeisis is normal in the absence of death machinery and have peripheral T cells that can mount anti-viral responses (Kaiser et al., 2014). However, the gross perturbations to the T cell compartment that occur in such mice in the absence of Fas signaling make it hard to assess whether T cell maturation is normal. Here, we observed a significant reduction in IL-7R expression following T-cell-specific deletion of RIPK1. The defect was more modest than in other strains that completely lacked NF-κB activity, suggesting that NF-κB activation downstream of TNFRSF signaling is not entirely RIPK1 dependent in thymocytes. However, the peripheral lymphopenia was as profound as other strains lacking NF-κB signaling. Our data suggest that thymocytes are weakly susceptible to TNF-induced cell death independently of RIPK1, possibly mediated by TNFR complex IIa. It is possible that the activity of this complex is enhanced as caspase-8 expression is upregulated in mature SP thymocyte subsets, which, in combination with reduced IL-7 survival signaling and other pro-survival NF-κB targets, results in the profoundly atrophied peripheral T cell compartment observed.

Our data reveal that RIPK1 expression is under developmental regulation in T cells, reaching peak abundance only in the most mature thymic populations. While RIPK1 is a critical component of the necroptosome, thymocytes do not express MLKL and are therefore not susceptible to necroptosis. Consistent with this, caspase-8-deficient T cells only become susceptible to necroptosis after TCR triggering (Ch'en et al., 2011). Taken together, this suggests that death signaling in T cells is developmentally regulated. In thymocytes, RIPK1 expression is induced, but at this stage its physiological role is to transmit TNFRSF signals required for normal NF-κB activation and maturation of new T cells. It is not until T cells become activated that RIPK1 can additionally mediate host defense functions by triggering necroptosis in susceptible cells, for instance during viral infections (Mocarski et al., 2011). Whether RIPK1 continues to facilitate transmission of TNFRSF-derived signals that are important in development of effector and memory responses will be an important area of future research.

Finally, expression of kinase-dead RIPK1^{D138N} *in vivo* resulted in a near-complete rescue of IKK-deficient CD4 SP thymocytes but only a partial rescue of mature CD8 SP thymocytes. While RIPK1 is one target of IKK responsible for regulating survival of mature CD8^{+} SP thymocytes, these data suggest that the IKK complex may control survival of these cells by other mechanisms independent of both RIPK1 and NF-κB. Our data reveal that thymocytes are only modestly sensitive to TNF-induced death in the absence of RIPK1 expression, whereas IKK deficiency renders them exquisitely sensitive to TNF-induced death, underscoring a critical role of IKKs in keeping RIPK1-dependent cell death in check. It may be relevant that TNFR1 deficiency also fails to completely rescue survival of mature, IKK-deficient CD8^{+} SP thymocytes (Webb et al., 2016). This could argue for the involvement of other death receptors, such as DR3, in regulating thymocyte survival. However, given the complexity of the downstream signalosome responsible for TNFR signaling, together with the developmental changes in gene expression of key signaling components such as RIPK1 and caspase-8, it is possible that removal of one or more components disturbs the fine equilibrium regulating formation of the different signaling complexes, which results in spontaneous, ligand-independent signaling. Whatever the explanation, it is clear that regulation of CD8^{+} SP thymocyte survival is complex. Nevertheless, identifying the importance of NF-κB-independent functions of the IKK complex for thymocyte survival and specifically the role of the IKK-RIPK1 pathway will greatly facilitate future studies of IKK-dependent cell survival.

STAR★METHODS

Detailed methods are provided in the online version of this paper and include the following:

- KEY RESOURCES TABLE
- CONTACT FOR REAGENT AND RESOURCE SHARING
- EXPERIMENTAL MODEL AND SUBJECT DETAILS
- METHOD DETAILS
  - Flow Cytometry
  - *In Vitro* Culture
  - RNA-Seq Analysis
  - Immunoblotting and Complex I Immunoprecipitation
- QUANTIFICATION AND STATISTICAL ANALYSIS
- DATA AND SOFTWARE AVAILABILITY

SUPPLEMENTAL INFORMATION

Supplemental Information includes one figure and can be found with this article online at https://doi.org/10.1016/j.immuni.2019.01.004.

ACKNOWLEDGEMENTS

We thank UCL Comparative Biology Unit staff for assistance with mouse breeding and maintenance, Janani Sivakumaran-Nguyen from the UCL Institute of Immunity and Transplantation Flow Cytometry Facility for help performing cell sorting, and Paola Niola from UCL Genomics for performing RNA sequencing and analysis. We thank Dr. Joe Grove, Dr. Alessandro Annibaldi, and Professor Pascal Meier for technical assistance and generous sharing of reagents. We thank the following for generous sharing of their mouse strains: Professor Manolis Pasparakis for the *Chuk* conditional strain,

Professor Michael Karin for the *Ikbkb* conditional strain, Professor Vishva Dixit for the RIPK1^{D138N} strain, Professor Albert Baldwin for the *Rela* conditional strain, and Steve Gerondakis for *Rel*$^{-/-}$ mice. Grant support in the Vandenabeele group included: Belgian (Interuniversity Attraction Poles: IAP 7/32, EOS 30826052 MODEL-IDI), Flemish (Research Foundation Flanders: FWO G.0875.11, G.0973.11, G.0A45.12N, G.0787.13N, G.0C31.14N, G.0C37.14N, G.0E04.16N, G.0C76.18N, and G.0B71.18N), a Methusalem grant (BOF16/MET_V/007, for N.T.), Ghent University (MRP, GROUP-ID consortium), the Foundation against Cancer (2012-188 and FAF-F/2016/865) and grants from VIB. J.H. has a FWO-SB PhD fellowship, and P.V. is senior full professor at UGent and PI at VIB. The work in the Seddon lab is supported by the Medical Research Council UK under program codes MC_PC_13055 and MR/P011225/1.

AUTHOR CONTRIBUTIONS

L.V.W., A.B., J.H., S.L., and B.S. designed experiments. L.V.W., A.B., J.H., and T.V.B. performed experiments. B.S. wrote the manuscript. J.H., T.V.B., N.T., and P.V. performed critical reading of the manuscript. L.V.W., A.B., and B.S. edited the manuscript.

DECLARATION OF INTERESTS

The authors declare no competing interests.

Received: March 19, 2018
Revised: November 7, 2018
Accepted: January 4, 2019
Published: February 5, 2019

REFERENCES

Annibaldi, A., and Meier, P. (2018). Checkpoints in TNF-Induced Cell Death: Implications in Inflammation and Cancer. Trends Mol. Med. *24*, 49–65.

Bonizzi, G., and Karin, M. (2004). The two NF-kappaB activation pathways and their role in innate and adaptive immunity. Trends Immunol. *25*, 280–288.

Ch'en, I.L., Tsau, J.S., Molkentin, J.D., Komatsu, M., and Hedrick, S.M. (2011). Mechanisms of necroptosis in T cells. J. Exp. Med. *208*, 633–641.

Chen, X., Willette-Brown, J., Wu, X., Hu, Y., Howard, O.M., Hu, Y., and Oppenheim, J.J. (2015). IKKa is required for the homeostasis of regulatory T cells and for the expansion of both regulatory and effector CD4 T cells. FASEB J. *29*, 443–454.

Clark, K., Peggie, M., Plater, L., Sorcek, R.J., Young, E.R., Madwed, J.B., Hough, J., McIver, E.G., and Cohen, P. (2011). Novel cross-talk within the IKK family controls innate immunity. Biochem. J. *434*, 93–104.

de Boer, J., Williams, A., Skavdis, G., Harker, N., Coles, M., Tolaini, M., Norton, T., Williams, K., Roderick, K., Potocnik, A.J., and Kioussis, D. (2003). Transgenic mice with hematopoietic and lymphoid specific expression of Cre. Eur. J. Immunol. *33*, 314–325.

Dillon, C.P., Weinlich, R., Rodriguez, D.A., Cripps, J.G., Quarato, G., Gurung, P., Verbist, K.C., Brewer, T.L., Llambi, F., Gong, Y.-N., et al. (2014). RIPK1 blocks early postnatal lethality mediated by caspase-8 and RIPK3. Cell *157*, 1189–1202.

Dondelinger, Y., Aguileta, M.A., Goossens, V., Dubuisson, C., Grootjans, S., Dejardin, E., Vandenabeele, P., and Bertrand, M.J. (2013). RIPK3 contributes to TNFR1-mediated RIPK1 kinase-dependent apoptosis in conditions of cIAP1/2 depletion or TAK1 kinase inhibition. Cell Death Differ. *20*, 1381–1392.

Dondelinger, Y., Jouan-Lanhouet, S., Divert, T., Theatre, E., Bertin, J., Gough, P.J., Giansanti, P., Heck, A.J., Dejardin, E., Vandenabeele, P., and Bertrand, M.J. (2015). NF-κB-Independent Role of IKKα/IKKβ in Preventing RIPK1 Kinase-Dependent Apoptotic and Necroptotic Cell Death during TNF Signaling. Mol. Cell *60*, 63–76.

Dondelinger, Y., Vandenabeele, P., and Bertrand, M.J. (2016). Regulation of RIPK1's cell death function by phosphorylation. Cell Cycle *15*, 5–6.

Gareus, R., Huth, M., Breiden, B., Nenci, A., Rösch, N., Haase, I., Bloch, W., Sandhoff, K., and Pasparakis, M. (2007). Normal epidermal differentiation but impaired skin-barrier formation upon keratinocyte-restricted IKK1 ablation. Nat. Cell Biol. *9*, 461–469.

Geng, J., Ito, Y., Shi, L., Amin, P., Chu, J., Ouchida, A.T., Mookhtiar, A.K., Zhao, H., Xu, D., Shan, B., et al. (2017). Regulation of RIPK1 activation by TAK1-mediated phosphorylation dictates apoptosis and necroptosis. Nat. Commun. *8*, 359.

Gugasyan, R., Horat, E., Kinkel, S.A., Ross, F., Grigoriadis, G., Gray, D., O'Keeffe, M., Berzins, S.P., Belz, G.T., Grumont, R.J., et al. (2012). The NF-κB1 transcription factor prevents the intrathymic development of CD8 T cells with memory properties. EMBO J. *31*, 692–706.

Hettmann, T., and Leiden, J.M. (2000). NF-kappa B is required for the positive selection of CD8+ thymocytes. J. Immunol. *165*, 5004–5010.

Isomura, I., Palmer, S., Grumont, R.J., Bunting, K., Hoyne, G., Wilkinson, N., Banerjee, A., Proietto, A., Gugasyan, R., Wu, L., et al. (2009). c-Rel is required for the development of thymic Foxp3+ CD4 regulatory T cells. J. Exp. Med. *206*, 3001–3014.

Jimi, E., Strickland, I., Voll, R.E., Long, M., and Ghosh, S. (2008). Differential role of the transcription factor NF-kappaB in selection and survival of CD4+ and CD8+ thymocytes. Immunity *29*, 523–537.

Kaiser, W.J., Daley-Bauer, L.P., Thapa, R.J., Mandal, P., Berger, S.B., Huang, C., Sundararajan, A., Guo, H., Roback, L., Speck, S.H., et al. (2014). RIP1 suppresses innate immune necrotic as well as apoptotic cell death during mammalian parturition. Proc. Natl. Acad. Sci. USA *111*, 7753–7758.

Kelliher, M.A., Grimm, S., Ishida, Y., Kuo, F., Stanger, B.Z., and Leder, P. (1998). The death domain kinase RIP mediates the TNF-induced NF-kappaB signal. Immunity *8*, 297–303.

Legarda-Addison, D., Hase, H., O'Donnell, M.A., and Ting, A.T. (2009). NEMO/IKKgamma regulates an early NF-kappaB-independent cell-death checkpoint during TNF signaling. Cell Death Differ. *16*, 1279–1288.

Li, Z.W., Omori, S.A., Labuda, T., Karin, M., and Rickert, R.C. (2003). IKK beta is required for peripheral B cell survival and proliferation. J. Immunol. *170*, 4630–4637.

Liu, H.H., Xie, M., Schneider, M.D., and Chen, Z.J. (2006). Essential role of TAK1 in thymocyte development and activation. Proc. Natl. Acad. Sci. USA *103*, 11677–11682.

Micheau, O., and Tschopp, J. (2003). Induction of TNF receptor I-mediated apoptosis via two sequential signaling complexes. Cell *114*, 181–190.

Miller, M.L., Mashayekhi, M., Chen, L., Zhou, P., Liu, X., Michelotti, M., Tramontini Gunn, N., Powers, S., Zhu, X., Evaristo, C., et al. (2014). Basal NF-κB controls IL-7 responsiveness of quiescent naïve T cells. Proc. Natl. Acad. Sci. USA *111*, 7397–7402.

Mocarski, E.S., Upton, J.W., and Kaiser, W.J. (2011). Viral infection and the evolution of caspase 8-regulated apoptotic and necrotic death pathways. Nat. Rev. Immunol. *12*, 79–88.

Mora, A.L., Chen, D., Boothby, M., and Rubin, D.H. (1999). Lineage-specific differences among CD8+ T cells in their dependence of NF-kappa B/Rel signaling. Eur. J. Immunol. *29*, 2968–2980.

Newton, K., Dugger, D.L., Wickliffe, K.E., Kapoor, N., de Almagro, M.C., Vucic, D., Komuves, L., Ferrando, R.E., French, D.M., Webster, J., et al. (2014). Activity of protein kinase RIPK3 determines whether cells die by necroptosis or apoptosis. Science *343*, 1357–1360.

Pasparakis, M., and Vandenabeele, P. (2015). Necroptosis and its role in inflammation. Nature *517*, 311–320.

Rickard, J.A., O'Donnell, J.A., Evans, J.M., Lalaoui, N., Poh, A.R., Rogers, T., Vince, J.E., Lawlor, K.E., Ninnis, R.L., Anderton, H., et al. (2014). RIPK1 regulates RIPK3-MLKL-driven systemic inflammation and emergency hematopoiesis. Cell *157*, 1175–1188.

Schmidt-Supprian, M., Courtois, G., Tian, J., Coyle, A.J., Israël, A., Rajewsky, K., and Pasparakis, M. (2003). Mature T cells depend on signaling through the IKK complex. Immunity *19*, 377–389.

Schmidt-Supprian, M., Tian, J., Grant, E.P., Pasparakis, M., Maehr, R., Ovaa, H., Ploegh, H.L., Coyle, A.J., and Rajewsky, K. (2004). Differential dependence of CD4+CD25+ regulatory and natural killer-like T cells on signals leading to NF-kappaB activation. Proc. Natl. Acad. Sci. USA *101*, 4566–4571.

Silva, A., Cornish, G., Ley, S.C., and Seddon, B. (2014). NF-κB signaling mediates homeostatic maturation of new T cells. Proc. Natl. Acad. Sci. USA *111*, E846–E855.

Sinclair, C., Saini, M., Schim van der Loeff, I., Sakaguchi, S., and Seddon, B. (2011). The long-term survival potential of mature T lymphocytes is programmed during development in the thymus. Sci. Signal. *4*, ra77.

Sinclair, C., Ono, M., and Seddon, B. (2015). A Zap70-dependent feedback circuit is essential for efficient selection of CD4 lineage thymocytes. Immunol. Cell Biol. *93*, 406–416.

Srinivas, S., Watanabe, T., Lin, C.S., William, C.M., Tanabe, Y., Jessell, T.M., and Costantini, F. (2001). Cre reporter strains produced by targeted insertion of EYFP and ECFP into the ROSA26 locus. BMC Dev. Biol. *1*, 4.

Teh, C.E., Lalaoui, N., Jain, R., Policheni, A.N., Heinlein, M., Alvarez-Diaz, S., Sheridan, J.M., Rieser, E., Deuser, S., Darding, M., et al. (2016). Linear ubiquitin chain assembly complex coordinates late thymic T-cell differentiation and regulatory T-cell homeostasis. Nat. Commun. *7*, 13353.

Ting, A.T., and Bertrand, M.J.M. (2016). More to Life than NF-κB in TNFR1 Signaling. Trends Immunol. *37*, 535–545.

Vandenabeele, P., Declercq, W., Van Herreweghe, F., and Vanden Berghe, T. (2010a). The role of the kinases RIP1 and RIP3 in TNF-induced necrosis. Sci. Signal. *3*, re4.

Vandenabeele, P., Galluzzi, L., Vanden Berghe, T., and Kroemer, G. (2010b). Molecular mechanisms of necroptosis: an ordered cellular explosion. Nat. Rev. Mol. Cell Biol. *11*, 700–714.

Voll, R.E., Jimi, E., Phillips, R.J., Barber, D.F., Rincon, M., Hayday, A.C., Flavell, R.A., and Ghosh, S. (2000). NF-kappa B activation by the pre-T cell receptor serves as a selective survival signal in T lymphocyte development. Immunity *13*, 677–689.

Waelchli, R., Bollbuck, B., Bruns, C., Buhl, T., Eder, J., Feifel, R., Hersperger, R., Janser, P., Revesz, L., Zerwes, H.G., and Schlapbach, A. (2006). Design and preparation of 2-benzamido-pyrimidines as inhibitors of IKK. Bioorg. Med. Chem. Lett. *16*, 108–112.

Wan, Y.Y., Chi, H., Xie, M., Schneider, M.D., and Flavell, R.A. (2006). The kinase TAK1 integrates antigen and cytokine receptor signaling for T cell development, survival and function. Nat. Immunol. *7*, 851–858.

Wang, L., Du, F., and Wang, X. (2008). TNF-alpha induces two distinct caspase-8 activation pathways. Cell *133*, 693–703.

Webb, L.V., Ley, S.C., and Seddon, B. (2016). TNF activation of NF-κB is essential for development of single-positive thymocytes. J. Exp. Med. *213*, 1399–1407.

Xing, Y., Wang, X., Jameson, S.C., and Hogquist, K.A. (2016). Late stages of T cell maturation in the thymus involve NF-κB and tonic type I interferon signaling. Nat. Immunol. *17*, 565–573.

Xu, C., Wu, X., Zhang, X., Xie, Q., Fan, C., and Zhang, H. (2018). Embryonic Lethality and Host Immunity of RelA-Deficient Mice Are Mediated by Both Apoptosis and Necroptosis. J. Immunol. *200*, 271–285.

Zheng, Y., Vig, M., Lyons, J., Van Parijs, L., and Beg, A.A. (2003). Combined deficiency of p50 and cRel in CD4+ T cells reveals an essential requirement for nuclear factor kappaB in regulating mature T cell survival and in vivo function. J. Exp. Med. *197*, 861–874.

STAR★METHODS

KEY RESOURCES TABLE

REAGENT or RESOURCE	SOURCE	IDENTIFIER
Antibodies		
CD3	BD Biosciences	Cat# 553058; RRID: AB_394591
CD28	BD Biosciences	Cat# 553295; RRID: AB_394764
PE-IL-7Rα	Thermo Fisher Scientific	Cat# 12-1273-81; RRID: AB_953566
CD4-PE	BioLegend	Cat# 116006; RRID: AB_313691
CD4-BV650	BioLegend	Cat# 100555; RRID: AB_2562529
CD4-E450	Thermo Fisher Scientific	Cat# 48-0042-82; RRID: AB_1272194
CD5-BV510	BioLegend	Cat# 100627; RRID: AB_2563930
CD5-APC	Thermo Fisher Scientific	Cat# 17-0051-82; RRID: AB_469331
TCR-PE-Cy5	BioLegend	Cat# 306710; RRID: AB_314648
TCR-PerCPcy5.5	Tonbo Biosciences	Cat# 65-5961; RRID: AB_2621911
CD44-APC-Cy7	BioLegend	Cat# 103028; RRID: AB_830785
CD44-BUV737	BD Biosciences	Cat# 564392; RRID: AB_2738785
CD44-BV786	BioLegend	Cat# 103059; RRID: AB_2571953
CD62L-APC	BioLegend	Cat# 104412; RRID: AB_313099
CD8-APC	BioLegend	Cat# 140410; RRID: AB_10641696
CD8-BUV395	BD Biosciences	Cat# 563786; RRID: AB_2732919
CD8-BV786	BioLegend	Cat# 344739; RRID: AB_2566201
CD25-PE-Cy7	Thermo Fisher Scientific	Cat# 25-0251-82; RRID: AB_469608
CD24-BUV737	BD Biosciences	Cat# 565308; RRID: AB_2739174
PE-Cy7-CD24	Thermo Fisher Scientific	Cat# 25-0242-82; RRID: AB_10853806
RIP (D94C12) XP Rabbit mAb antibody	Cell Signaling Technology	Cat# 3493; RRID: AB_2305314
Rabbit IgG Isotype Control Monoclonal Antibody, Isotype Control, Unconjugated, Clone DA1E	Cell Signaling Technology	Cat# 3900; RRID: AB_1550038
Anti-rabbit IgG (H+L), F(ab)2 Fragment (Alexa Fluor 647 Conjugate) antibody	Cell Signaling Technology	Cat# 4414S; RRID: AB_10693544
Chemicals, Peptides, and Recombinant Proteins		
Recombinant mouse TNF	Peprotech	Cat# 315-01A
3XFLAG-mTNF		Prof. Pascal Meier
IKK2 inhibitor BI605906		Prof. Philip Cohen
IKK16	Selleck	Cat# 52882
Necrostatin 1	Santa Cruz Biotech	Cat# SC-200142
FLAG M2 affinity gel	Sigma-Aldrich	Cat# A2220; RRID: AB_10063035
USP2	Bio-Techne	Cat# E504
Lambda protein phosphatase	New England BioLabs	Cat# P07535
CTV	Life Technologies	Cat# C34557
Critical Commercial Assays		
NuPage 3%–8% Bis-Tris gel	Life Technologies	EA 0375
Deposited Data		
RNA sequencing analysis of *Tnfr1a*$^{-/-}$ IKK-deficient CD8SP thymocytes versus WT	ArrayExpress	E-MTAB-4778
RNA sequencing analysis of CD8SP thymocytes from WT and *CD4*^{Cre} *Rela*$^{fx/fx}$ *Rel*$^{-/-}$ *Nfkb1*$^{-/-}$ mice	ArrayExpress	TBC
Experimental Models: Organisms/Strains		
C57Bl6/J	UCL Comparative Biology Unit	Strain 0159
Chuk$^{fx/fx}$		Prof. Manolis Pasparakis

(*Continued on next page*)

Continued		
REAGENT or RESOURCE	SOURCE	IDENTIFIER
Ikbkb$^{fx/fx}$		Prof. Michael Karin
Ripk1^{D138N}		Prof. Vishva Dixit
Rela$^{fx/fx}$		Prof. Albert Baldwin
Rel$^{-/-}$		Prof. Steve Gerondakis
Nfkb1$^{-/-}$		Prof. Steve Gerondakis
Ripk1$^{fx/fx}$		Prof. Peter Vandenabeele
CD4^{Cre}	The Jackson Laboratory	Stock No: 017336
huCD2^{iCre}	MRC National Institute for Medical Research	Dr. Dimitris Kioussis
Tnfrsf1a$^{-/-}$	The Jackson Laboratory	Stock No. 002818
Chuk$^{fx/fx}$ *Ikbkb*$^{fx/fx}$ *R26*^{REYFP} *huCD2*^{iCre}	UCL Comparative Biology Unit	This study
Chuk$^{fx/fx}$ *R26*^{REYFP} *huCD2*^{iCre}	UCL Comparative Biology Unit	This study
Chuk$^{fx/fx}$ *Ikbkb*$^{fx/fx}$ *R26*^{REYFP} *CD4*^{cre}	UCL Comparative Biology Unit	This study
Tnfrsf1a$^{-/-}$ *Chuk*$^{fx/fx}$ *Ikbkb*$^{fx/fx}$ *R26*^{REYFP} *CD4cre*	UCL Comparative Biology Unit	This study
Ripk1^{D138N} *Chuk*$^{fx/fx}$ *Ikbkb*$^{fx/fx}$ *R26*^{REYFP} *huCD2*^{iCre}	UCL Comparative Biology Unit	This study
Ripk1$^{fx/fx}$ *CD4*^{cre}	VIB-UGent Center for Inflammation Research	This study
Software and Algorithms		
FlowJo Software	FlowJo LLC	N/A
GraphPad Prism	GraphPad Software, Inc	N/A

CONTACT FOR REAGENT AND RESOURCE SHARING

Further information and requests for resources and reagents should be directed to and will be fulfilled by the Lead Contact, Benedict Seddon (benedict.seddon@ucl.ac.uk).

EXPERIMENTAL MODEL AND SUBJECT DETAILS

Mice with conditional alleles of *Ikbkb* (*Ikbkb*$^{fx/fx}$) (Li et al., 2003) and *Chuk* (*Chuk*$^{fx/fx}$) (Gareus et al., 2007) were intercrossed with mice either expressing transgenic *cre* under the control of the human *Cd2* (ΔT^{huCD2}) (de Boer et al., 2003) or *Cd4* expression elements (ΔT^{CD4}). *Rosa26* reporter YFP allele (R26^{REYFP}) (Srinivas et al., 2001) was also used, in order to facilitate identification of cells in which *cre* recombinase had been active, while *Cd4*^{Cre} strains were additionally backcrossed onto a *Tnfrsf1a*$^{-/-}$ background. Compound mutants of *Rela*, *Nfkb1* and *Rel* were generated by intercrossing *Nfkb1*$^{-/-}$, *Rel*$^{-/-}$ and *Rela*$^{fx/fx}$ *Cd4*^{Cre} strains. *Chuk*$^{fx/fx}$ *Ikbkb*$^{fx/fx}$ *R26*^{REYFP} *huCD2*^{iCre}, *Chuk*$^{fx/fx}$ *R26*^{REYFP} *huCD2*^{iCre}, *Chuk*$^{fx/fx}$ *Ikk2*$^{fx/fx}$ *R26*^{REYFP} *Cd4*^{Cre}, *Tnfrsf1a*$^{-/-}$ *Chuk*$^{fx/fx}$ *Ikbkb*$^{fx/fx}$ *R26*^{REYFP} *Cd4*^{Cre} and *Ripk1*^{D138N} *Chuk*$^{fx/fx}$ *Ikbkb*$^{fx/fx}$ *R26*^{REYFP} *huCD2*^{iCre}, mice were bred in the Comparative Biology Unit of the Royal Free UCL campus and at Charles River laboratories, Manston, UK. *Ripk1*$^{fx/fx}$ *Cd4*^{Cre} mice were breed at VIB. Ex vivo analysis of lymphoid organs was performed using mice between 8-12 weeks age unless otherwise specified. All mice are on a C57BL6/J background. Animal experiments were performed according to the UCL Animal Welfare and Ethical Review Body and Home Office regulations.

METHOD DETAILS

Flow Cytometry

Flow cytometric analysis was performed with 2-5 x 10^6 thymocytes, 1-5 x 10^6 lymph node or spleen cells. Cell concentrations of thymocytes, lymph node and spleen cells were determined with a Scharf Instruments Casy Counter. Cells were incubated with saturating concentrations of antibodies in 100 μl of phosphate-buffered saline (PBS) containing bovine serum albumin (BSA, 0.1%) and 1 mM azide (PBS-BSA-azide) for 45 min at 4°C followed by two washes in PBS-BSA-azide. Phycoerythrin (PE)–conjugated antibody against IL-7Rα, EF450-conjugated antibodies against CD4, APC-conjugated antibody against CD5, PE-Cy5– and PerCPcy5.5-conjugated antibody against TCR (H57-597), APC-Cy7– and APC-conjugated antibody against CD44, pacific orange (PO)–conjugated antibody against CD8, PE-Cy7- conjugated antibody against CD25, PE-Cy7-conjugated antibody against HSA(CD24) were purchased from eBioscience. RIPK1 was detect using 1μg/ml of anti-RIPK1 rabbit monoclonal (D94C12, Cell Signaling Technology) or isotype control (DA1E, Cell Signaling Technology) and a Alexa Fluor 647 conjugated secondary anti-Rabbit (Cell Signaling Technology). Cell samples were fixed in 1% PFA for 20 mins at 4°C and permeabilised with 0.1% NP40 for 3 mins prior to staining. Cell

viability was determined using LIVE/DEAD cell stain kit (Invitrogen Molecular Probes). Eight-color flow cytometric staining was analyzed on a FACSCanto II or LSRFortessa X-20 (Becton Dickinson) instrument. For cell sorting, lymphocytes were incubated with the appropriate antibodies for detection of surface markers and were then purified to >95% purity by high-speed sorting on an FACSAria flow cytometer (Becton Dickinson). Data analysis and colour compensations were performed with FlowJo V9.5.3 software (TreeStar). Live FSc versus SSc gates were defined following singlet gating on the basis of FSC-A versus FSC-W. Specific thymocyte populations were gated in the following manner. DP thymocytes – $CD4^+$ $CD8^+$, $CD4^+$ SP thymocytes – $CD4^+$ $CD8^-$ TCR^{hi}, $CD8^+$ SP thymocytes – $CD4^+$ $CD8^-$ TCR^{hi}. Peripheral populations in lymph node and spleen were gated as follows. $CD4^+$ T cells – $CD4^+$ $CD8^-$ TCR^{hi}, $CD8^+$ T cells – $CD4^+$ CD8- TCR^{hi}, Naive $CD4^+$ T cell – $CD4^+$ TCR^{hi} $CD44^{lo}$ $Foxp3^-$, Naive $CD8^+$ T cell – $CD8^+$ TCR^{hi} $CD44^{lo}$, $CD4^+$ memory – $CD4^+$ TCR^{hi} $CD44^{hi}$ $Foxp3^-$, Treg - $CD4^+$ TCR^{hi} $Foxp3^+$. Data are displayed on log and biexponential displays of fluorescence intensity in arbitrary units. Axis labels indicates the target protein to which fluorophore labelled antibodies are specific and fluorescent signal is display. Numbers on plots indicate % of cells falling in the corresponding gate.

In Vitro Culture

Thymocytes were cultured at 37oC with 5% CO2 in RPMI-1640 (Gibco, Invitrogen Corporation, CA) supplemented with 10% (v/v) fetal bovine serum (FBS) (Gibco Invitrogen), 0.1% (v/v) 2-mercaptoethanol βME (Sigma Aldrich) and 1% (v/v) penicillin-streptomycin (Gibco Invitrogen) (RPMI-10). Recombinant TNF was supplemented to cultures at 10ng/ml, unless otherwise stated, and was obtained from R&D, with PBS used as vehicle. Inhibitors were used at the following concentrations, unless otherwise stated : specific IKK2 inhibitor BI605906 (10μM in 0.1% DMSO vehicle), pan IKK inhibitor IKK16 (2μM in 0.1% DMSO), Necrostatin 1 (1μM in 0.1% DMSO). Cells were activated by CD3 (10μg/ml) and CD28 (10μg/ml) mAb bound to 96 well flat bottom plates overnight at 4°C and washed with PBS prior to culture. Cells were cultured at 10^6/ml in 200μl. TCR^{hi} $CD4^+$ SP and $CD8^+$ SP thymocytes were isolated by cell sorting from total thymocytes, labelled with CTV and cultured at 10^6/ml for 72hr in either CD3+CD28 mAb coated plates or uncoated wells as control. Cultures of peripheral T cells were performed by labelling total lymph node cell suspensions with CTV and culturing cells at 10^6/ml in 96 well flat bottom plates.

RNA-Seq Analysis

TCR^{hi} $CD8^+$ SP thymocytes were sorted from WT (n = 8) and $RelA^{\Delta T}$ *Nfkb1*$^{-/-}$ *Rel*$^{-/-}$ mice (n = 5), mRNA purified and gene expression determined by RNAseq analysis. Individual RNAseq libraries were generated from independent cell preparations. RNA was isolated from single cell suspensions using the RNeasy Mini Kit (Qiagen) according to the manufacturer's instructions. RNA integrity was confirmed using Agilent's 2200 Tapestation. Samples were processed using the SMART-Seq v4 Ultra Low Input RNA Kit (Clontech Laboratories, Inc.). Briefly, cDNA libraries were generated using the SMART (Switching Mechanism at 5' End of RNA Template) technology which produces full-length PCR amplified cDNA starting from 10ng total RNA. The amplified cDNA was checked for integrity and quantity on the Agilent Bioanalyser using the High Sensitivity DNA kit. 150pg of cDNA was then converted to sequencing library using the Nextera XT DNA (Illumina, San Diego, US). This uses a transposon able to fragment and tag the double-stranded cDNA (Tagmentation), followed by a limited PCR reaction (12 cycles). Libraries to be multiplexed in the same run are pooled in equimolar quantities, calculated from Qubit and Tapestation fragment analysis. Samples were sequenced on the NextSeq 500 instrument (Illumina, San Diego, US) using a 43bp paired end run.

Run data were demultiplexed and converted to fastq files using Illumina's bcl2fastq Conversion Software v2.19. Fastq files are pre-processed to remove adapter contamination and poor quality sequences (trimmomatic v0.36) before being mapped to a suitable reference genome using the spliced aligner STAR (v2.5b). Mapped data is deduplicated using Picard Tools (v2.7.1), in order to remove reads that are the result of PCR amplification, and remaining reads per transcript are counted by FeatureCounts (v1.4.6p5). Normalisation, modelling and differential expression analysis are then carried out using SARTools (v1.3.2), an integrated QC and DESeq2 BioConductor wrapper. After normalization, reads were displayed as reads per kilobase of exon per million reads (RPKM).

Immunoblotting and Complex I Immunoprecipitation

3 x 10^7 total thymocytes were used per condition. Where indicated, cells were pretreated for 30 min with Nec1 and/or IKK16 inhibitors. For complex I immunoprecipitations (IPs), cells were stimulated with 2 mg/ml 3xFLAG-TNF. Cells were washed two times in ice-cold PBS before lysis in 1 ml of NP-40 lysis buffer (10% glycerol, 1% NP-40, 150 mM NaCl, and 10 mM Tris-HCl [pH 8] supplemented with phosphatase and protease inhibitor cocktail tablets [Roche Diagnostics]). The cell lysates were cleared by centrifugation for 15 min at 40C, and the supernatant was then incubated overnight with FLAG M2 affinity gel at 4°C. The next day, the beads were washed three times in PBS buffer. The beads were then either resuspended in 10μl Laemmli buffer to elute the immune complexes or resuspended in 40μl of DUB/IPP buffer (50 mM Tris-HCl [pH 8], 50 mM NaCl, 5 mM DTT, and 1 mM MnCl2) to remove conjugated ubiquitin chains and phosphorylations on RIPK1. Then, either 1.8 mg USP2 or 800 U Lambda protein phosphatase (New England BioLabs) was added as indicated. Reactions were incubated for 30 min at 30 C and subsequently 30 min at 37°C. IPs and total cell extract loading controls were analyzed by NuPage 3%–8% Bis-Tris gel (Invitrogen Novex), transferred onto PVDF membrane (Millipore) and immunoblotted with anti-RIPK1 (Cell Signaling Technology). Immunodetection was performed by incubation with horseradish peroxidise-conjugated anti-rabbit (1:5000) (DAKO) and developed by enhanced chemiluminescence (Millipore).

QUANTIFICATION AND STATISTICAL ANALYSIS

Statistical analysis and figure preparation were performed using Graphpad Prism 6 (v6.0a). Two-way comparisons were made by nonparametric unpaired two-tailed Mann-Witney Student's t test while dose response curves and variation in cell numbers between strains over time were analyzed by two-way ANOVA. $^{*}p < 0.05$, $^{**}p < 0.01$, $^{***}p < 0.001$. Error bars indicate SD unless otherwise stated.

DATA AND SOFTWARE AVAILABILITY

The accession numbers for the data reported in this paper are ArrayExpress: E-MTAB-4778 and E-MTAB-7470.

Immunity, Volume *50*

Supplemental Information

Survival of Single Positive Thymocytes Depends upon Developmental Control of RIPK1 Kinase Signaling by the IKK Complex Independent of NF-κB

Louise V. Webb, Alessandro Barbarulo, Jelle Huysentruyt, Tom Vanden Berghe, Nozomi Takahashi, Steven Ley, Peter Vandenabeele, and Benedict Seddon

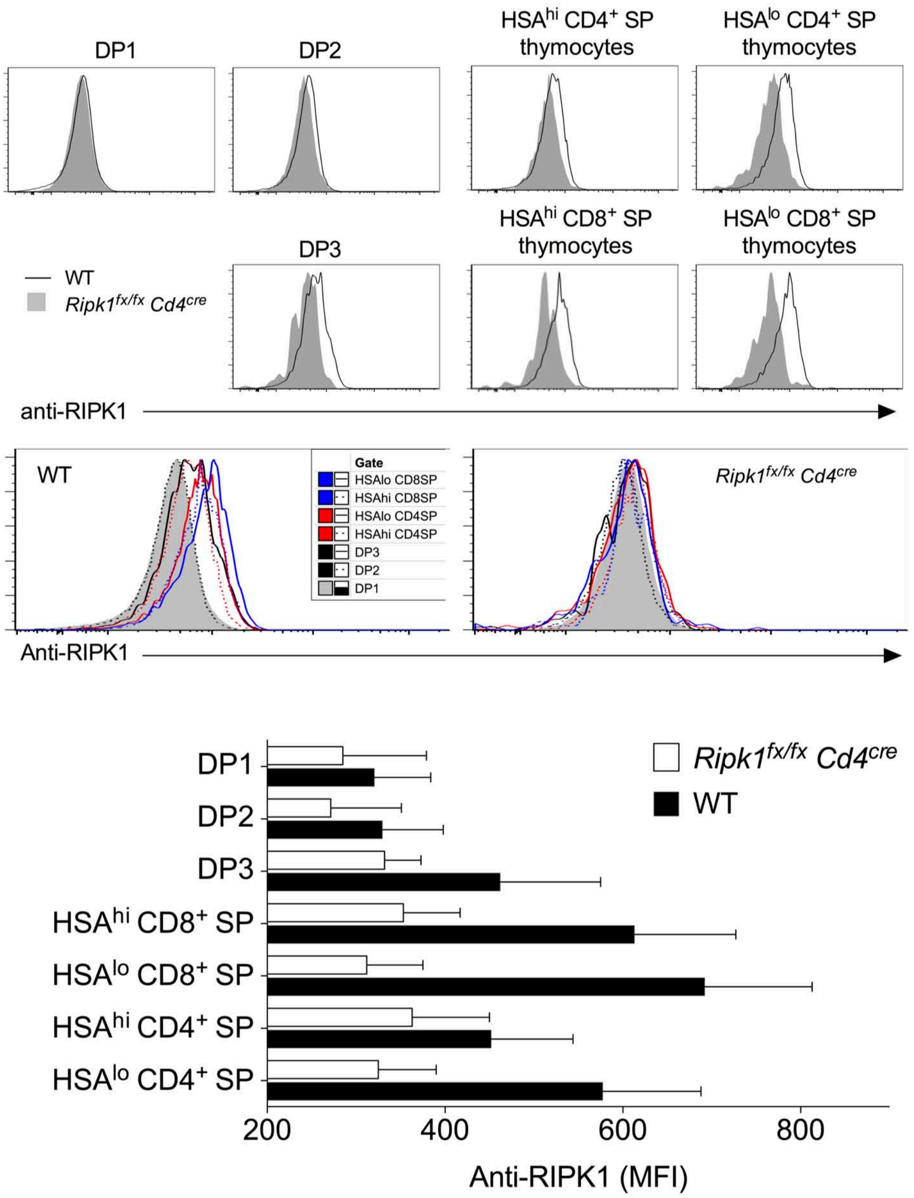

Figure S1 - RIPK1 expression in WT controls vs RIPK1 deficient thymocytes. Related to Figure 4. Thymocytes from 8 week old CD4^{Cre} *Ripk1*$^{fx/fx}$ (n=3) and Cre– littermates (n=3) were isolated and stained for expression of CD4, CD8, TCR, CD5, HSA followed by fixation and intracellular stain for RIPK1 expression. Histograms show RIPK1 staining by thymocytes from Cre+ (RIPK1 KO) and Cre- (WT), comparing either RIPK1 staining of specific thymic subsets between Cre+ and Cre- mice (top two rows), or directly comparing staining of different subpopulations from Cre– (WT) and CD4^{Cre} *Ripk1*$^{fx/fx}$ mice (bottom row). Subsets were gated as follows : DP1 (CD4$^+$CD8$^+$ TCR^{LO}CD5^{LO}), DP2 (CD4$^+$CD8$^+$ TCR^{INT}CD5^{HI}), DP3 (CD4$^+$CD8$^+$ TCR^{HI} CD5^{INT}), HSA^{hi} CD4SP (CD4$^+$CD8$^-$ TCR^{HI}CD5^{HI} HSA^{HI}), HSA^{lo} CD4SP (CD4$^+$CD8$^-$ TCR^{HI} CD5^{HI} HSA^{LO}), HSA^{hi} CD8SP (CD4$^-$CD8$^+$ TCR^{HI}CD5^{HI} HSA^{HI}) and HSA^{lo} CD8SP (CD4$^-$CD8$^+$ TCR^{HI}CD5^{HI} HSA^{LO}). Bar chart shows mean fluorescence intensity of RIPK1 staining on the indicated thymic subsets from either CD4^{Cre} *Ripk1*$^{fx/fx}$ (RIPK1 KO) and Cre– (WT) littermates.
